# Biophysics and electrophysiology of pulsed field ablation in normal and infarcted porcine cardiac ventricular tissue

**DOI:** 10.1038/s41598-024-83683-y

**Published:** 2024-12-30

**Authors:** Damijan Miklavčič, Atul Verma, Philippa R. P. Krahn, Jernej Štublar, Bor Kos, Terenz Escartin, Peter Lombergar, Nicolas Coulombe, Maria Terricabras, Tomaž Jarm, Matej Kranjc, Jennifer Barry, Lars Mattison, Nicole Kirchhof, Daniel C. Sigg, Mark Stewart, Graham Wright

**Affiliations:** 1 Faculty of Electrical Engineering, University of Ljubljana, Trzaska 25, Ljubljana, Slovenia; 2 McGill University Health Centre, McGill University, Montreal, Canada; 3 Sunnybrook Research Institute, Toronto, Canada; 4 Department of Cardiology Cardiovascular Surgery, University Clinical Medical Centre, Ljubljana, Slovenia; 5 Medtronic, Minneapolis, MN USA; 6 Department of Medical Biophysics, University of Toronto, Toronto, Canada

**Keywords:** Biomedical engineering, Interventional cardiology

## Abstract

Pulsed Field Ablation (PFA) is a new ablation method being rapidly adopted for treatment of atrial fibrillation, which shows advantages in safety and efficiency over radiofrequency and cryo-ablation. In this study, we used an in vivo swine model (10 healthy and 5 with chronic myocardial infarct) for ventricular PFA, collecting intracardiac electrograms, electro-anatomical maps, native T1-weighted and late gadolinium enhancement MRI, gross pathology, and histology. We used 1000–1500 V pulses, with 1–16 pulse trains to vary PFA dose. Lesions were assessed at 24 h, 7 days, and 6 weeks in healthy and at 48 h in infarcted ventricles. Comparisons of lesion sizes using a numerical model enabled us to determine lethal electric field thresholds for cardiac tissue and its dependence on the number of pulse trains. Similar thresholds were found in normal and infarcted hearts. Numerical modeling and temperature-sensitive MRI confirmed the nonthermal nature of PFA, with less than 2% of a lesion’s volume at the highest dose used being attributed to thermal damage. Longitudinal cardiac MRI and histology provide a comprehensive description of lesion maturation. Lesions shrink between 24 h and 7 days post-ablation and then remain stable out to 6 weeks post-ablation. Periprocedural electrograms analysis yields good correlation with lesion durability and size.

**Supplementary Information:**

The online version contains supplementary material available at 10.1038/s41598-024-83683-y. Data used to prepare the figures is available at https://doi.org/10.6084/m9.figshare.25894936.

## Introduction

Following the successful introduction of Pulsed Field Ablation (PFA) for the isolation of the pulmonary veins in the treatment of atrial fibrillation, by irreversible electroporation^1–4^, together with evidence of improved safety profiles and efficacy relative to radiofrequency ablation, there are now expanding efforts to use PFA for ventricular ablation^5–9^. A central challenge of ventricular ablation is creating deeper lesions in a thicker myocardium^10,11^. Furthermore, thermal ablative energies are limited in effectively ablating cardiac tissue with ischemic and nonischemic scars (which are often a cause of ventricular arrhythmias), due to poor thermal conductivity of scar tissue. By contrast, scarred myocardium has higher electric conductivity than normal myocardium^12–16^, which should facilitate electrical field penetration during PFA. Achieving larger and deeper lesions by PFA in thicker ventricular walls compared to atrial walls will require further optimization and/or adjustments to PFA such as higher dosing (using higher voltages and/or more pulses) or different vectoring of the field^17–19^. Using higher dosing may however lead to thermal damage and increase the risk of collateral injury.

In this study, experimental results were compared to theoretical predictions based on numerical modeling. Electroporation of tissue relies on the electric field causing cell membrane electroporation^20–22^. The lethal electric field threshold (LET) needed to achieve irreversible electroporation of cells depends on the specific waveform applied^23–25^, but is independent of electrode geometry and applied voltage. In contrast, the electric field distribution in tissue depends on the electrode geometry and the tissue’s electric properties^26,27^. Realizing favorable electric field distributions and LET needed to achieve durable deep lesions is thus a central focus of the current work.

Numerical modeling is a standard and reliable method for determination of electric field distribution in complex structures, and has recently been further developed and successfully used for studies in living tissues^25,28,29^. We thus used previously developed and validated numerical models of electric field distribution which take into account tissue heterogeneity, anisotropy, and the non-linear response of tissue when exposed to high-voltage electrical pulses^25,30–32^. These models of electric field distribution, together with prior knowledge of lesion creation in the myocardium, were used to determine the effects of dose by adjusting the LET based on known relationships between the dose and the LET^33^. Numerical modeling based on catheter position reconstruction also allowed us to assess the temperatures in targeted and collateral tissue (myocardium, blood, and vascular tissue in the inferior vena cava) during PFA deliveries.

Here, to determine for the first time the lethal electric field levels in vivo in healthy and in chronically infarcted pig hearts, we performed in vivo experiments using a focal PFA catheter. We assessed the time course of lesion development in healthy animals using intracardiac electrograms (iEGMs) and serial cardiac MRI (cMRI) at 24 h, 7 days and 6 weeks, with histopathology at the termination of experiments. In addition, electroanatomical mapping (EAM) and multi-view fluoroscopy combined with numerical modeling allowed us to compute the amplitude of the LET in cardiac tissue. Based on these results, we sought to identify early predictors of chronic lesion size. We demonstrated that durable lesions (visible on histology at 6 weeks post ablation) were achieved in the thick LV wall by focal PFA in this clinically relevant, large animal model and that lesion extent was dosedependent. Next, we showed that over the 6-week time course of lesion development the results of the iEGM and of the serial cMRI correlated well with lesion sizes in the hearts when determined via gross pathology and with lesion character (necrosis, fibrosis) when assessed on histology. Finally, PFA was performed in an infarcted pig model, which allowed us to test whether PFA could be used predictably in heterogenous scarred myocardium. Here the animals were sacrificed 48 h post-ablation.

## Results

Pulsed Field Ablation (PFA) was first performed in different segments of the left ventricle (LV) of healthy pigs using a bipolar two-catheter configuration, specifically between a focal ablation catheter and a return catheter placed in the inferior vena cava (IVC). The dose of PFA was titrated by changing the pulse amplitude (4-pulse trains at 1000, 1300 and 1500 V) and the number of pulse trains delivered (1-, 4-, 8- and 16-pulse trains at 1500 V). The selected dosing was chosen to create various PFA lesion sizes ranging from no durable lesion to durable transmural lesions, while preserving the nonthermal nature of PFA. Characteristics of lesions created by PFA in healthy animals were determined by cMRI at 24 h, 7 days, and 6 weeks using native T1-weighted (T1w) and late gadolinium enhancement (LGE) cMRI, as well as by gross pathology on formalin-fixed cardiac cross sections and histopathology at 6 weeks. We also recorded iEGMs continuously for 30 s before and up to 5 min post ablation at each location and analyzed PFA-induced iEGM changes which were then correlated to lesion size. Finally, PFA was performed in healthy LV and in scarred LV of pigs 4–6 weeks post-infarct to explore the capacity to create lesions in scarred myocardium; these PFA lesions were only assessed acutely, with sacrifice at 48 h.

### Delineation between thermal and nonthermal origin of lesion

**Fig. 1:**
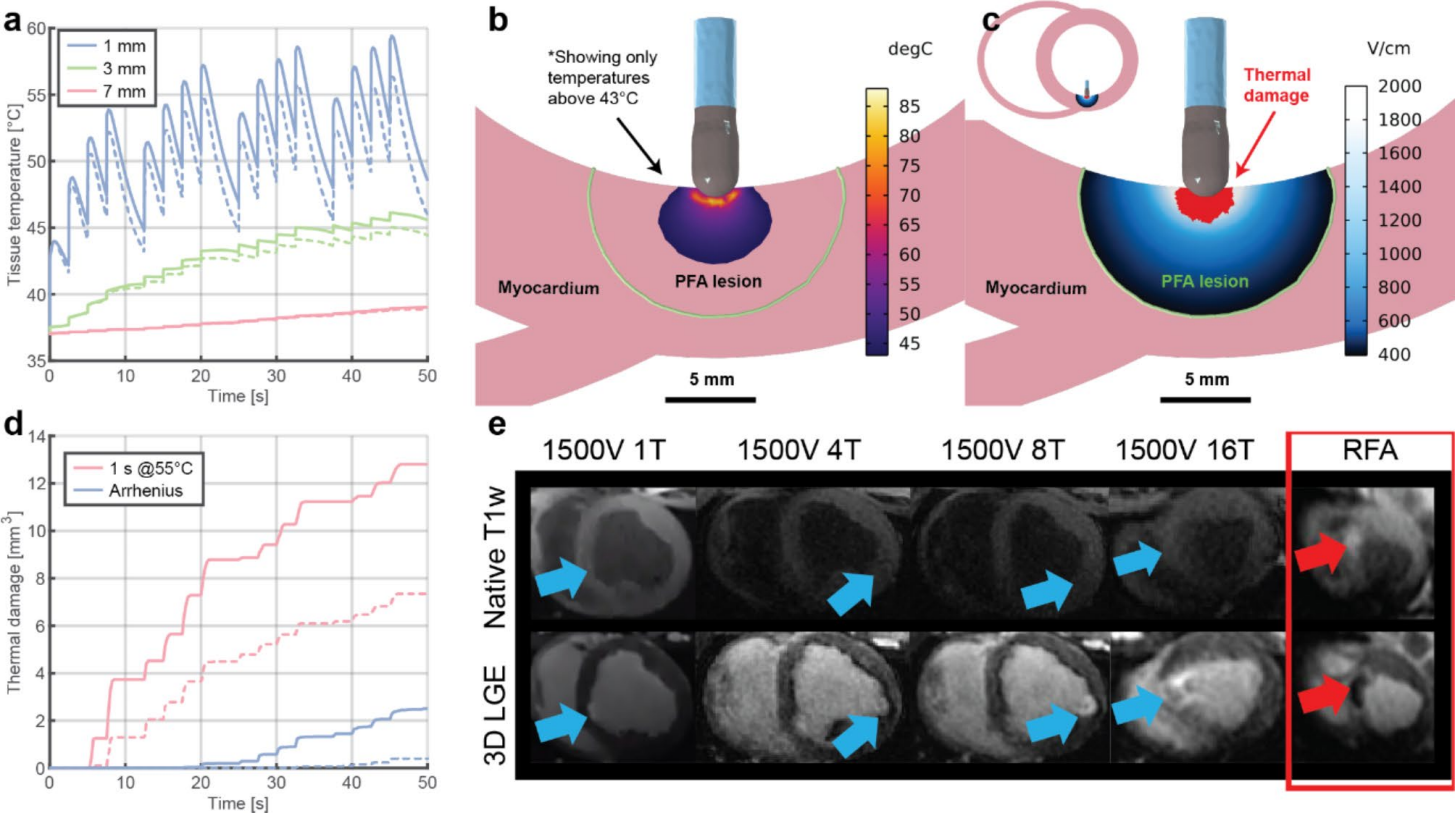
Nonthermal vs. thermal damage by Pulsed Field Ablation. (**a**) Calculated temperature in the myocardium at depths of 1, 3 and 7 mm for the 16-pulse-train protocol. Solid lines indicate low blood flow scenario and dashed lines indicate high flow scenario. (**b**) Calculated temperature distribution in the myocardium at the end of the 16th train (peak temperature). Only temperatures above 43 °C are shown. Black line indicates possible size of the PFA lesion based on the 16-train 7-day MRI LET (median value: 394 V/cm, Fig. 6b). Grid size is 1 × 1 mm. (**c**) Predicted nonthermal vs. thermal damage for 16-train protocol. Calculated electric field distribution in the myocardium indicates the PFA lesion based on the 16-train 7-day MRI LET – only electric field values above 394 V/cm are shown. Red area indicates possible thermal damage using a threshold of 1 s at ≥ 55 °C in low blood-flow condition (worst case). (**d**) Calculated thermal damage for 16 train protocol using the threshold of 1 s at ≥ 55 °C and Arrhenius integral - probability of cell death exceeding 63%. Solid lines indicate low blood flow scenario and dashed lines indicate high flow scenario. All temperature and thermal damage calculations shown here were done for the mid posterior lesion, for which the model predicts the highest electric current. (**e**) Native T1w and 3D LGE MRI at different PFA doses at 24 h post-ablation (blue arrows), in comparison to same acquisitions for RFA (red arrows and red frame). Native T1w PFA images show absence of hyperintensity at lesion locations suggesting lethal thermal threshold was not achieved, compared with clear hyperintense lesion in RFA indicating lethal thermal damage. 3D LGE cMRI demonstrates hyperintensity in PFA locations despite the absence of hyperintensity in native T1w with some microvascular obstruction (MVO) structure at highest PFA dose, while image of RFA lesion shows large MVO. RFA – Radiofrequency ablation; LGE – Late gadolinium enhancement; PFA - Pulsed field ablation; T- train.

Delivery of highvoltage electric pulses (i.e., PFA) with the aim of creating a durable lesion in myocardium by irreversible electroporation inevitably results in some Joule heating. This heating is greatest closest to the electrodes on the catheter. We used numerical modeling to estimate heating in the vicinity of the ablation catheter with two blood flow conditions, which are important as blood flow affects the rate of cooling of the overheated tissue (see Methods, subsection Numerical modeling – temperature and thermal damage). The maximum computed temperatures at 1, 3, and 7 mm depth under the ablation catheter were 60, 46, and 39 °C, respectively (Fig. 1a). The maximum predicted volume of thermal damage was 13 mm^3^ (representing less than 2% of the total lesion volume) using a threshold of 1 s at ≥ 55 °C in a low blood-flow condition (Fig. 1d). The maximal volume of tissue with a probability of cell death exceeding 63% was 2.5 mm^3^ using Arrhenius integral in a low blood-flow condition (Fig. 1d). Figure 1b and c illustrate the extent of maximal thermal damage based on the numerical model in comparison with the total lesion size achieved by PFA – because of irreversible electroporation. The minute volumes of thermal damage obtained with the numerical models are consistent with the lack of a thermal signature in iron-sensitive native T1w MR. This finding contrasts greatly with the imaging after a thermal RF ablation treatment (Fig. 1e). Numerical modeling also showed that peak temperatures on the electrode-blood surface of the ablation catheter did not exceed 66 °C in a low blood-flow condition, while the temperatures on electrode-blood surface of the return catheter in IVC did not exceed 41 °C in a worst-case scenario, specifically, a low blood-flow condition and with the return catheter pressed against the IVC wall.

### PFA Lesion development dynamics using MRI

**Fig. 2:**
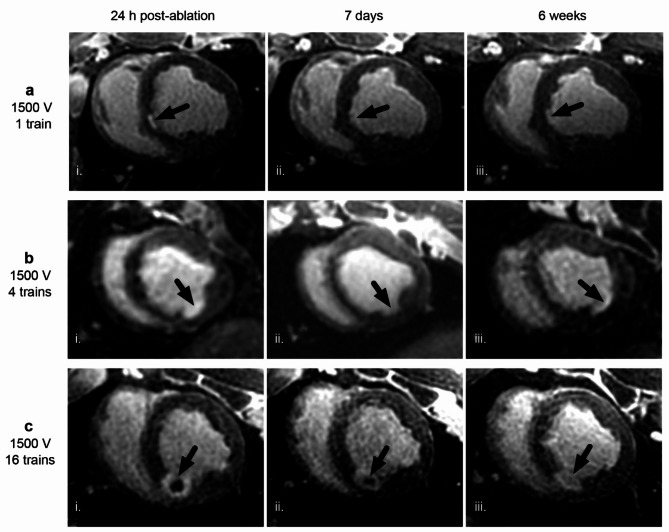
3D LGE cMR images of PFA lesions (arrows) at 24 h, 7 days, and 6 weeks after ablation of healthy porcine LV myocardium. PFA lesions shown were created in the (**a**) septum, (**b**) inferior wall, and (**c**) posterior wall using differing numbers of pulse trains, all at 1500 V. All images were acquired 15 min after gadolinium injection. The lesion created using the highest PFA dose c) shows a central dark region with slow penetration of gadolinium, likely associated with microvascular obstruction (MVO). At 24 h post-ablation all doses (a-i, b-i and c-i) show hyperintense regions indicative of increased interstitial fluid, vascular permeability and permeability of irreversibly damaged cells, which are diminished at later time points (ii and iii), consistent with edema resolution and degradation of dead cells.

**Fig. 3:**
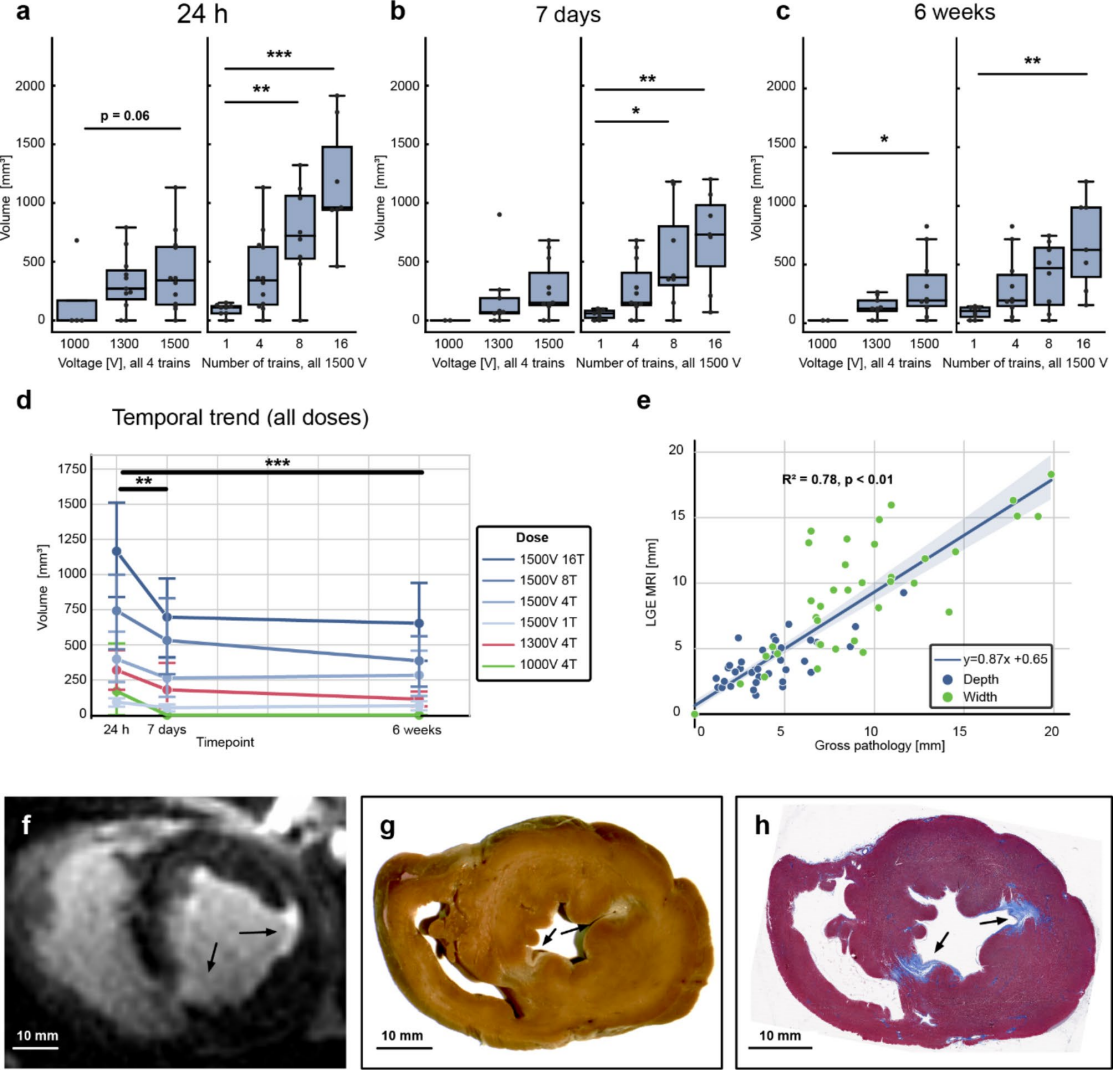
Dose-dependence of lesion volume measured using LGE cMRI. Data at (**a**) 24 h, (**b**) 7 days, and (**c**) 6 weeks after ablation. A linear mixed effects model was used to compare voltages, taking the form: volume ~ voltage + (1|ID) (*p < 0.05, **p < 0.01, ***p < 0.001). Similarly, the model used to compare varying numbers of pulse trains at 1500 V was: volume ~ Ntrains + (1|ID). (**d**) Temporal evolution of lesion volumes created by different PFA dose schemes (model: volume ~ time + (1|ID) + (1|voltage) + (1|Ntrains)). (**e**). Strong agreement was observed between the 6-week lesion dimensions measured using LGE cMRI and gross pathology. Taken separately, the depth and width models were y = 0.65x + 1.18 (R^2^ = 0.59, *p* < 0.01), and y = 0.81x + 1.86 (R^2^ = 0.66 *p* < 0.01) respectively. PFA lesions at 6 weeks visualized in f) 3D LGE cMRI (30 min after gadolinium injection), in g) a formalin-fixed cross section of the heart, and in h) a Masson’s trichrome stained histology slide (scale bars: 10 mm).

**Fig. 4:**
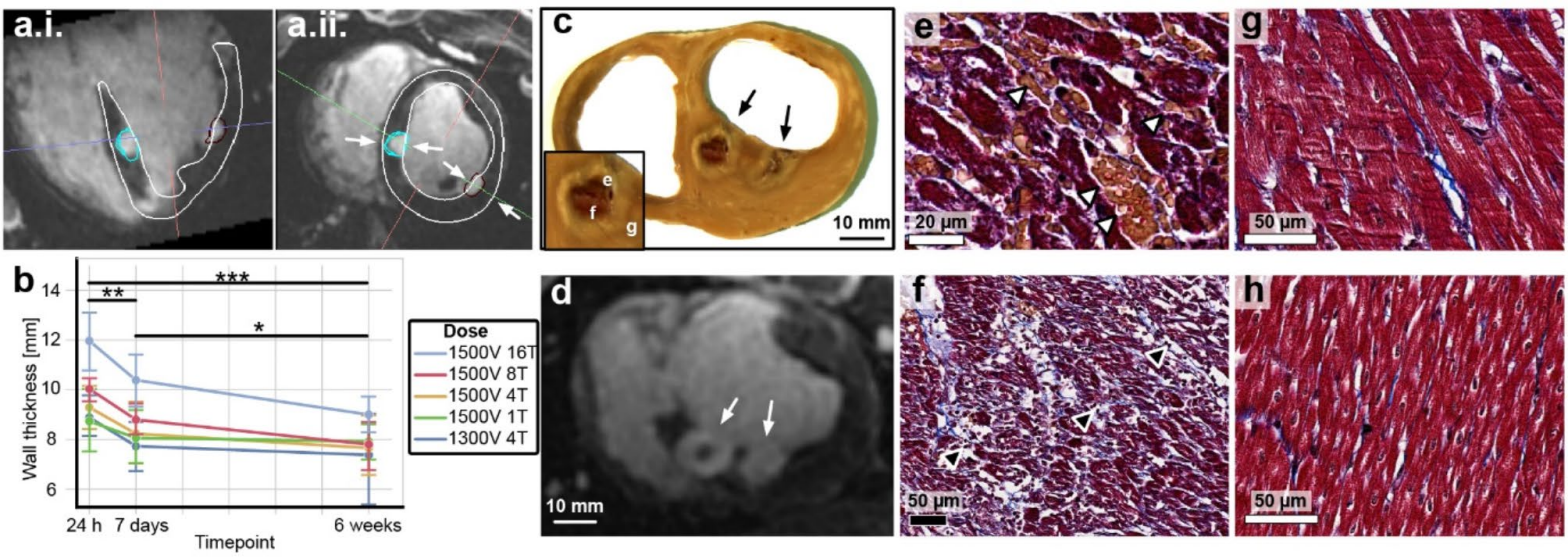
Evaluation of LV wall thickness and local tissue characteristics after PFA using cMRI and histology. (**a**) 3D LGE cMR images 24 h post ablation, formatted along long-axis (a.i.) and short-axis (a.ii.) planes used to measure LV wall thickness at the site of each ablation. (**b**) Summary of cMRI-derived LV wall thickness at the site of PFA using differing dose schemes (T = pulse trains) at 24 h, 7 days, and 6 weeks after ablation. A linear mixed effects model controlling for individual differences and number of pulse trains revealed that wall thickness was significantly higher at 24 h post-ablation compared to subsequent time points (*p < 0.05, **p < 0.01, ***p < 0.001). (**c**) Formalin-fixed cross section of a heart of a pig sacrificed at 48-h time point and (**d**) corresponding 3D LGE cMRI showing two adjacent 48-h old PFA lesions (arrows), both created by delivering 1500 V in 8-pulse trains. Masson’s trichrome stained sections from a 48-h time point showing (**e**) hemorrhage (arrowheads), (**f**) minor interstitial leukocytic infiltrates (arrowheads), and (g) contraction band necrosis. Healthy myocardium at a remote site H) is within normal limits.

Longitudinal observation of cardiac lesions by LGE cMRI provided a signature of tissue response to PFA not described previously. PFA lesions were visualized as enhanced signal intensity in LGE cMRI images on the LV endocardium. The signal reflected increased penetration and distribution of gadolinium with respect to surrounding healthy myocardium (Fig. 2) at 24 h. At this time point, the lesion volumes were the largest, as they subsequently decreased significantly within the next 7 days and from then on remained largely constant out to 6 weeks (Fig. 3d). Lesion volumes determined by LGE cMRI at 7 days and 6 weeks correlated well (Fig. 3e) with the lesion size determined in gross pathology at 6 weeks (Fig. 3g) and histological imaging (Fig. 3h). Lesion volumes as determined by LGE cMRI at 7 days could thus be used as an early predictor of chronic lesion size. The observed overestimation of lesion volumes at 24 h via LGE cMRI can be ascribed to increased vascular permeability leading to interstitial fluid accumulation (edema). Histology confirmed this diagnosis, and also showed acute cellular infiltration (Fig. 4 and Supplement Fig. 1 at 48 and 7 h post PFA, respectively) associated with a solid area of dead myocardium (cells with leaky plasma membrane in situ) that is removed within the first week by immune cells. While histology demonstrates the presence of replacement fibrosis in the lesion at 6 weeks, LV wall thickness at the lesion location remained similar to that of remote healthy myocardium on average, since most lesions are not transmural.

In a subset of 7/37 (19%) lesions at 24 h after PFA, a central core was observed with reduced signal intensity surrounded by a bright rim in LGE cMR images, (e.g., Fig. 2c). These were observed in dosing schemes: 1300 V 4-pulse-train (1 lesion), 1500 V 4-pulse-train (2), 1500 V 8-pulse-train (2), and 1500 V 16-pulse-train (2). This pattern of enhancement, also known as microvascular obstruction (MVO), is consistently observed with thermal lesions (e.g., RFA in Fig. 1e).

### Electrophysiology of PFA

**Fig. 5:**
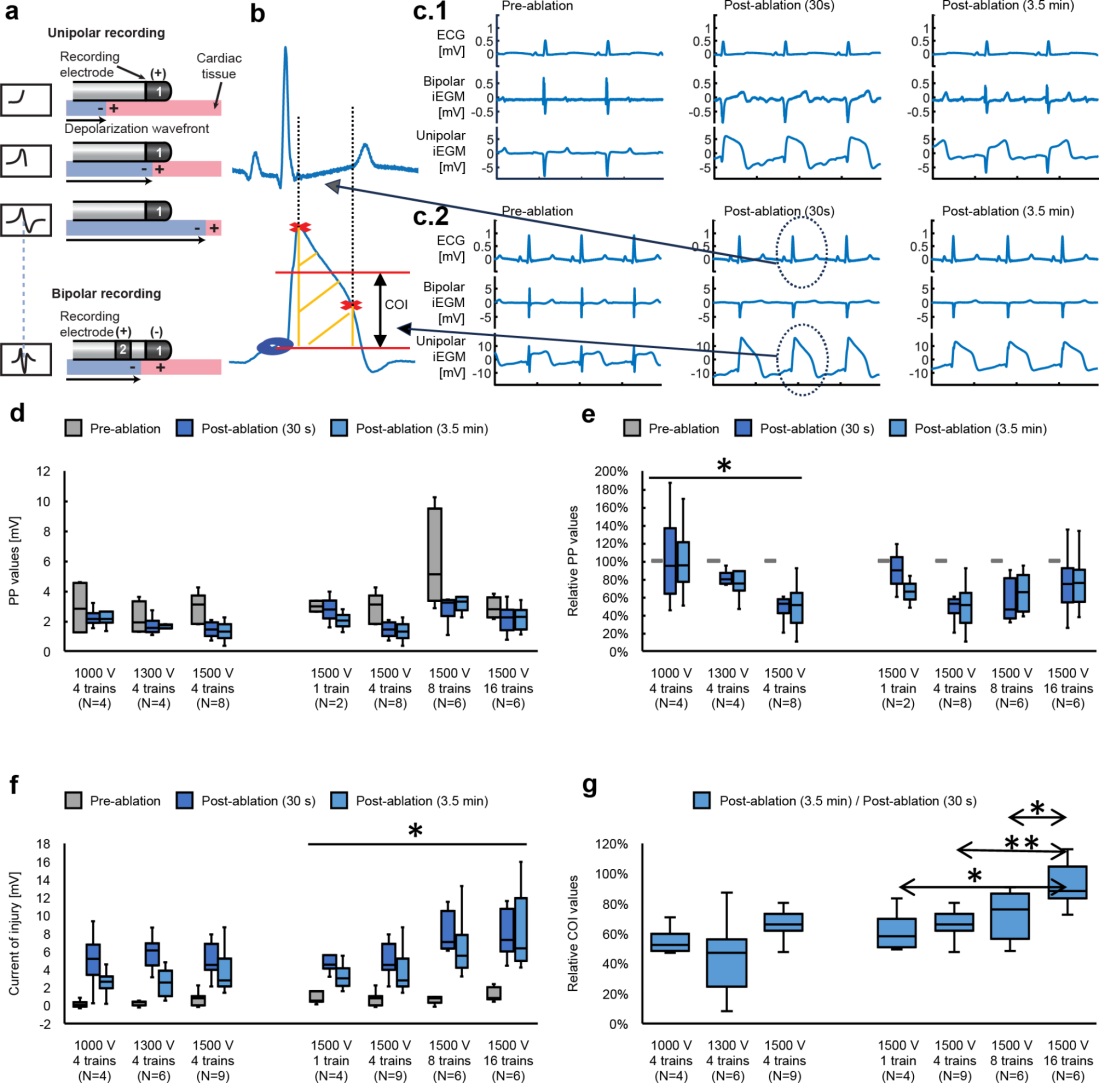
Evaluation of electrogram changes after PFA. (**a**) Schematic drawing of unipolar and bipolar iEGM recording generation. (**b**) Calculation of the current of injury (COI) parameter from unipolar iEGM signals. Blue circles: baseline value measured before the QRS. Red crosses: the start and the end of the area under the curve (AUC - shaded area) window, based on criteria described in the methods. The COI parameter (black arrow) was defined as the AUC divided by the AUC window width. (**c**) Examples of concrete signals recorded at two different ventricular sites treated with two different doses of PFA and shown for three different time points (pre-ablation, 30 s post-ablation and 3.5 min post-ablation). **(c.1**) Example 1: a lower dose (1300 V, 4 trains); (**c.2**) Example 2: a higher dose (1500 V, 8 trains). (**d**) and (**e**) Comparison of changes in peak-to-peak amplitude of bipolar iEGMs (bandwidth: 30–500 Hz). **(d**) Absolute values at three different time points (pre-ablation, 30 s post-ablation and 3.5 min post-ablation) grouped by the dose delivered. (**e**) Post-ablation values normalized to the pre-ablation values. A linear mixed effects (LME) model was used to compare doses and post-treatment times, taking the form: relative PP values ~ Time + Dose + (1|PigID/lesion) (*p < 0.05), where the dose was either the voltage or the number of pulses. (**f**) and **(g**): Comparison of changes in COI of unipolar iEGMs (bandwidth: 0.5–500 Hz). (**f**) Absolute values at three different time points (pre-ablation, 30 s post-ablation and 3.5 min post-ablation) grouped by the dose delivered. LME model was used to compare doses and post-treatment times, taking the form: COI ~ Time + Dose + (1|PigID/lesion) (*p < 0.05), where the dose was either the voltage or the number of pulses. (**g**) Values of COI recorded 3.5 min post-ablation normalized to COI values recorded 30 s post-ablation. LME model was used to compare doses, taking the form: relative COI ~ Dose + (1|PigID) (*p < 0.05, **p < 0.01), where the dose was either the voltage or the number of pulses.

We monitored bipolar iEGMs (with standard frequency bandwidth of 30–500 Hz, as often used clinically to guide thermal ablations) and unipolar iEGMs (with extended frequency bandwidth of 0.5–500 Hz) during PFA (Fig. 5a). The delivery of electric pulses resulted in profound morphological changes in bipolar and unipolar iEGMs (Fig. 5c). Delivery of PFA caused immediate reduction of the depolarization component of bipolar iEGM signals, similar to what is usually seen after thermal ablation. However, this reduction was accompanied by the appearance of a prominent downward spike (broader in appearance than the previous depolarization component) (Fig. 5c; compare the pre- and 30 s post-ablation bipolar iEGMs). In unipolar iEGMs, we consistently observed a current of injury (COI) phenomenon.

The two opposing effects resulted in smaller overall reductions in peak-to-peak values of bipolar iEGMs after PFA than is typically seen in thermal ablation, with little or no subsequent recovery observed within the first 3.5 min post ablation (compare 30 s and 3.5 min post-ablation bipolar iEGM signals in Fig. 5c.2). This reduction in overall peak-to-peak values for bipolar iEGMs and the lack of significant recovery from 30 s to 3.5 min post-ablation is summarized in Fig. 5d (absolute values) and Fig. 5e (post-ablation values normalized to the pre-treatment values). In Fig. 5d, the overall decrease of peak-to-peak values observed 30 s and 3.5 min post-ablation was always statistically significant (*p* < 0.05).

Conversely, the unipolar iEGM signals showed a rapid increase of the peak-to-peak value of the signal followed by a gradual reduction within the 3.5-min post-ablation observation period (Fig. 5c.1). The morphological change in unipolar iEGMs after PFA resembled the ST elevation which is likely a COI phenomenon. While the response of unipolar iEGMs to PFA also exhibited an immediate decrease in the depolarization component of the signal, similar to bipolar iEGMs, the appearance (or a large increase) of the COI phenomenon was the dominating feature in unipolar iEGMs changes after PFA. The quantification of the COI phenomenon is described in the methods section and illustrated in Fig. 5b. The overall increase in COI was statistically significant at all doses for both post-treatment intervals (*p* < 0.05). No overall dose dependence of COI on voltage for 4 pulse trains was observed (Fig. 5f left) while the dependence on the number of pulse trains at 1500 V (Fig. 5f right) was statistically significant (*p* < 0.05). In Fig. 5g, the 3.5-min post-ablation values normalized to the 30 s post-ablation values are shown for better visualization of the post-ablation recovery. The recovery dynamics of COI showed a clear dose dependence on the number of pulse trains at 1500 V. Faster recovery dynamics of COI was observed at lower doses.

COI evaluated from unipolar iEGMs at 3.5 min post-ablation as a potential intraprocedural index of pulsed field ablation was correlated to lesion sizes measured from gross pathology (at the chronic time point) or assessed from LGE cMRI (Supplemental Figure S2). The best correlation (R^2^ = 0.77) was observed for lesion volumes estimated from gross pathology measurements. In contrast, no correlation between the reduction of the bipolar iEGMs and lesion size was observed (Supplemental Figure S3).

### Lethal Electric Field threshold (LET) of cardiac tissue

**Fig. 6:**
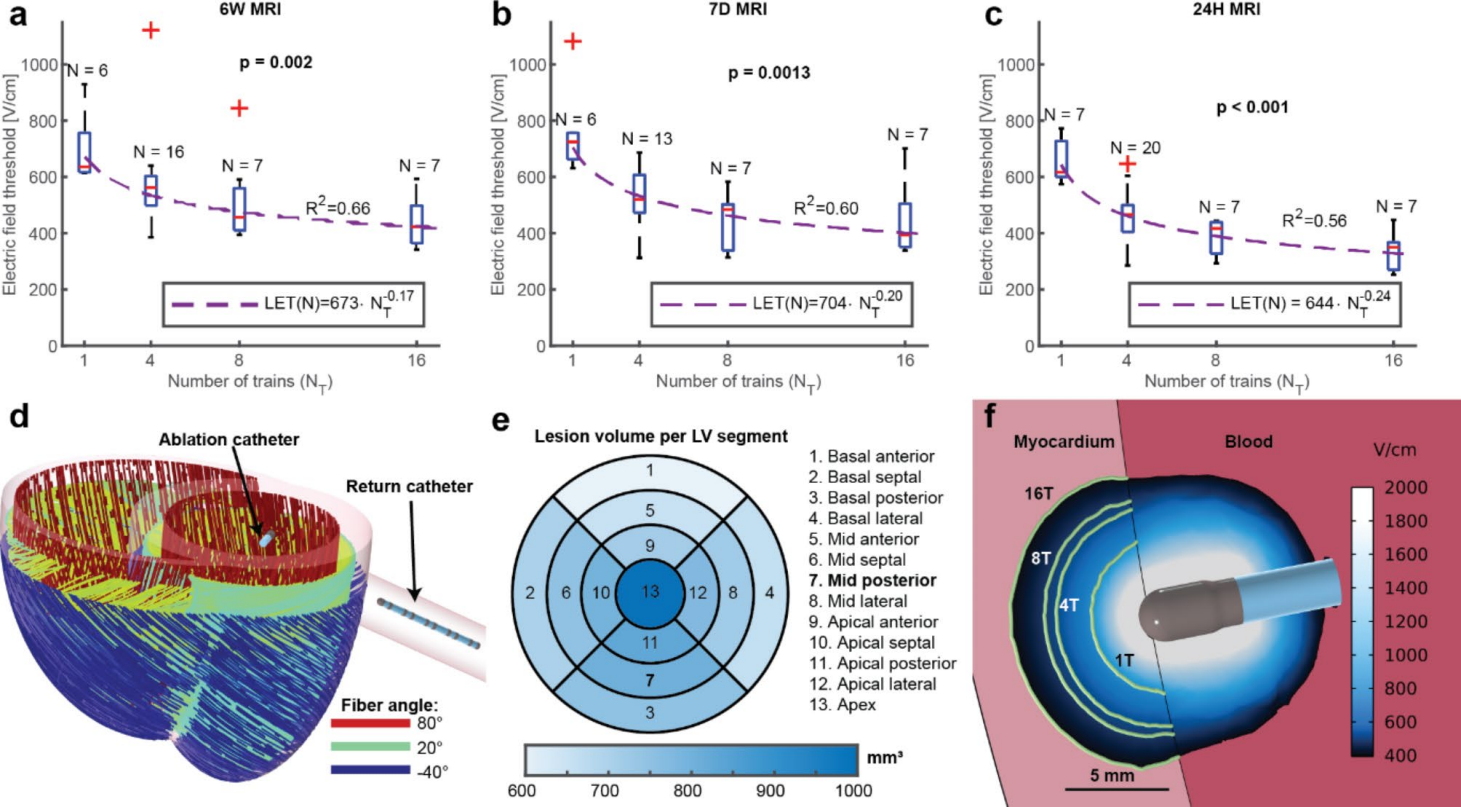
Lethal electric field threshold (LET). LET was calculated/obtained by comparison of numerical model and volume determined by 3D LGE cMRI at different time points after ablation and number of trains: (**a**) **24 h**: median LET was 617, 466, 417, and 350 V/cm for 1, 4, 8, and 16 trains, respectively. (**b**) **7 days**: median LET was 725, 520, 484, and 394 V/cm for 1, 4, 8, and 16-pulse-trains, respectively. (**c**) **6 weeks**: median LET was, was 636, 562, 457, and 423 V/cm for 1, 4, 8, and 16-pulse-trains, respectively. (**d**) 3D schematic biventricular geometry with the cardiac fiber orientation, which was used in the numerical model to calculate electric field distribution and determine LET. Image generated using COMSOL Multiphysics. (**e**) Predicted lesion volumes for lesions located in different LV segments based on the 16-pulse-train 7-day LGE cMRI LET (394 V/cm). (**f**) Predicted electric field distribution in the cardiac tissue and blood in the case of mid posterior lesion with the green contours representing the median LET threshold calculated with the 7-day LGE cMRI volumes for different number of trains as marked on the individual contours.

We were able to determine the lethal electric field threshold (LET) in vivo. This was done by using numerical modeling to calculate electric field distribution and determine lesion volume at a certain LET and comparing this with the lesion volume measured on 3D LGE cMRI. A matching LET was found for every experimental lesion at all available time points (Fig. 6a-c), taking into consideration the location of each individual lesion in healthy animals. A power function^33^ was fitted to the LET as a function of the number of trains for all three evaluation time points (Fig. 6a-c). The median LET for the 24 h time point was lower than for the 7-day and 6-week time points, which corresponds to larger LGE volumes seen at the earlier time point.

We can use these calculated LET to evaluate the impact of electrode geometries (e.g. distance between the catheters) on lesion size. In our study ablations were made by positioning the focal catheter in different segments of LV (basal, mid, apical, and anterior, septal, lateral, posterior). Due to varying distances between the ablation and return catheter (LV and IVC, respectively) and depending on where in the LV the ablation catheter was positioned, different lesion volumes were expected based on LET (Fig. 6e and Supplemental Figure S4); however, the catheter location within the LV was not significantly correlated with lesion size at any time point of the evaluation due to the large variability present in the data (*p* > 0.05, Spearman’s rho correlation coefficient).

We also determined the distribution of the electric field in blood, but we did not determine the LET for erythrocytes in this study. The LET for erythrocytes is higher than for cardiomyocytes because they are much smaller. The smaller size of erythrocytes leads to lower induced transmembrane voltages compared to larger cardiomyocytes at the same (extracellular) electric fields. Consequently, electroporation of erythrocytes membranes takes place at higher electric field strengths. The calculated distribution of the electric field in the blood (Fig. 6f) shows that the volume of blood exposed to an electric field above 1000 V/cm is 234 mm^3^, of which 177 mm^3^is in the LV and 57 mm^3^in the IVC. The volume of blood exposed to an electric field above 1500 V/cm was 101 mm^3^, of which 90 mm^3^in the LV and 11 mm^3^ in the IVC. These values are instantaneous volumes that do not take into account blood flow and should therefore be considered as the volume exposed to a single pulse train.

### Scarred tissue: real-life challenge of cardiac ablation

**Fig.7:**
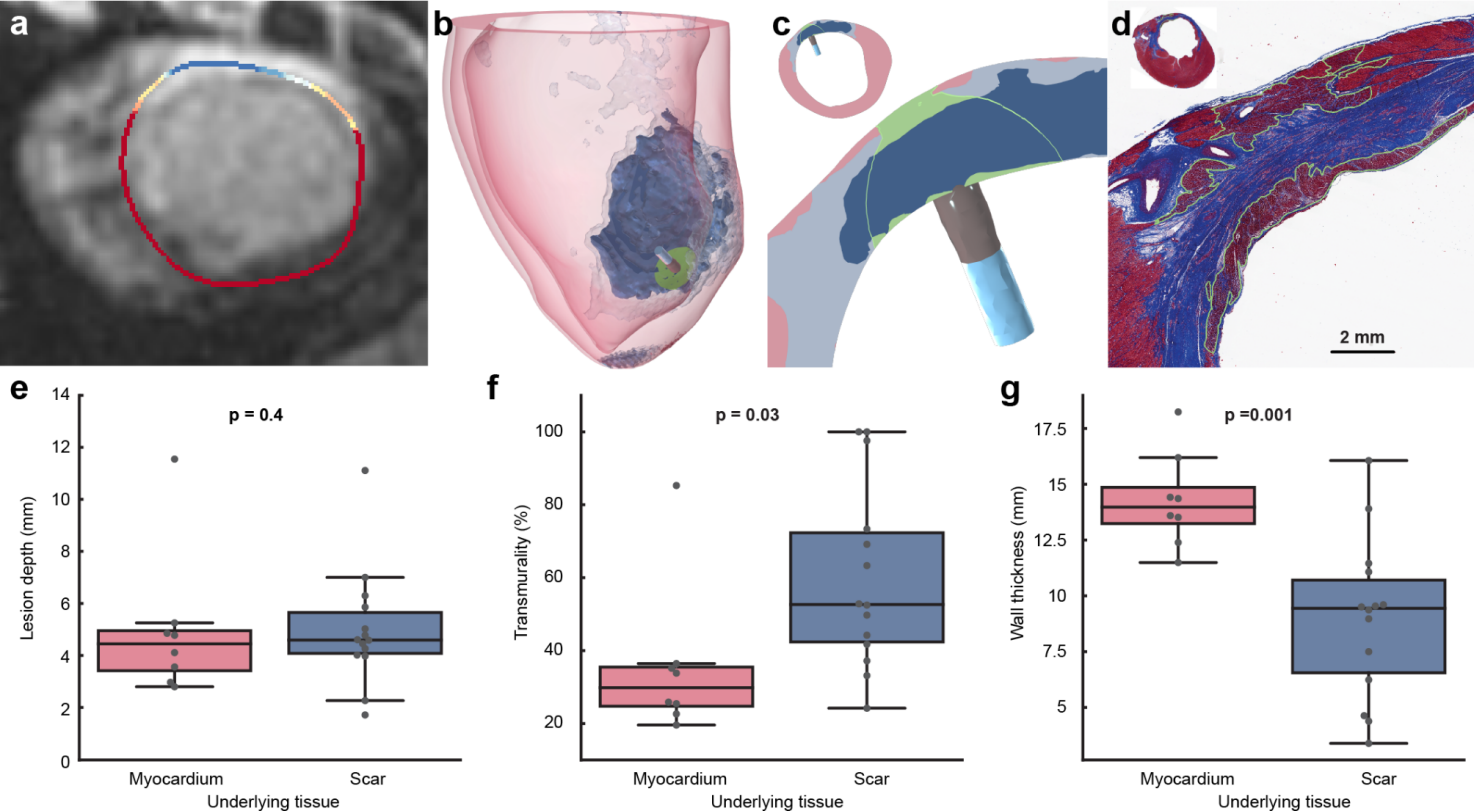
Pulsed field ablation delivered and simulated in infarcted ventricles. (**a**) Short-axis LGE cMRI showing infarct on the anterior side of the left ventricle. (**b**) 3D numerical model of scarred tissue. Healthy myocardium is shaded red, dense scar and border zone are dark and light blue respectively, and the PFA lesion is shaded in green. The catheter can be seen in the upper left part of the circled area. For PFA lesions, the LET determined based on MRI at 48 h (8 trains) for healthy tissue in infarcted pigs was chosen (median value: 456 V/cm, which is higher than the threshold determined in *healthy* animals at 24 h (417 V/cm), however the difference was not significant (*p* = 0.11, Kruskal-Wallis test)). The location of the image plane in a) and 2D view in (c) is indicated in blue. (**c**) Short axis cross-section of the LV at the lesion location. The image shows the in silico electric field above the LET, outlined in green. (**d**) The corresponding Masson’s trichrome stained histology section demonstrates the presence of an acute transmural PFA lesion despite the presence of significant regional intramural myocardial scar (red = myocardium, blue = fibrosis, purple = irreversible injury, acute lesion is outlined in green). (**e**) Comparison of depth, (**f**) comparison of transmurality, and (**g**) comparison of wall thickness of lesions created in healthy myocardium or scarred tissue, across the 5 infarcted animals (p-values calculated using LME models to control for individual differences).

In a series of chronically infarcted porcine hearts exhibiting dense fibrous remodeling (scarring) of the LV, we targeted the PFA (1500 V 8 trains) at either healthy myocardium, border zone, or dense scar via an endocardial approach. The objective was to investigate the capacity of PFA to create lesions in the presence of scarred tissue using the same bipolar catheter configuration, and to compare lesion extent against predictions from modelling. Four to six weeks after the infarction, we confirmed using LGE cMRI the presence of partly heterogeneous (mixture of viable myocardium and fibrosis), partly dense, nontransmural infarct zones in the LV, as expected for this porcine model. For the ablation procedure, we were guided by MRI maps of scar overlaid onto electro-anatomical maps (EAM) (Fig. 7). Areas of dense scar in the EAM were identified using < 0.5 mV cutoff bipolar voltage, with healthy myocardium identified as > 1.5 mV. Border zone areas between dense scar and healthy myocardium were defined with peak-to-peak voltage amplitudes between 0.5 and 1.5 mV, corresponding to areas of heterogeneous scar^34^. Current clinical substrate ablation strategies are based on voltage amplitude on EAM; electrogram characteristics are also used to locate any slow conducting, fibrous areas, called channels or corridors. LGE cMRI has recently become a useful tool to identify arrhythmia substrate and potential targets for ablation. Scar maps in LGE cMRI are constructed based on pixel signal intensity relative to maximum values, being < 40% in healthy myocardium, > 60% in dense scar and 40–60% in heterogeneous scar. Like in the cohort of healthy animals, we recorded iEGMs for 30 s before ablation and 5 min post ablation at each location.

All animals underwent cMRI and were euthanized approximately 48 h post PFA ablation. Evaluation of postmortem tissue via gross and histopathology allowed us to observe the early response to PFA in the 3 targeted ablation zones (i.e. healthy, border, scar). Lesion sizes were determined by LGE cMRI, from formalin fixed cardiac tissue slices, and on the corresponding histological sections. Histology specimens were stained with Masson’s trichrome to visualize the postinfarct fibrosis, and to facilitate the outline of the recent PFA ablation of included cardiomyocytes. We could accurately determine ablation catheter positioning and orientation relative to MRI and histology using multiple X-ray projections and electro-anatomical mapping. Numerical modeling of electric field distribution including tissue anisotropy, electrical conductivity changes due to electroporation and heterogeneous conductivity of the chronically infarcted heart model allowed us to show predicted lesion extent 48 h post PFA. A numerical model based on reconstructed endocardial position of the catheter during ablation (Fig. 7b) in a chronically infarcted region demonstrated that the electric field penetrates the dense scar located in the middle of the myocardium (Fig. 7c). In this chronically infarct swine model, the extent of the lesion formation towards the epicardial side beyond the intramural scar matched the observed extent of lesion formation on histopathology (Fig. 7f).

Among the 5 infarcted animals, lesions created in scarred underlying tissue (5.0 ± 2.2 mm, *N* = 14) were not significantly shallower than those created in healthy myocardium (5.0 ± 2. mm, *N* = 8, *p* = 0.40) (Fig. 7d). However, transmurality was higher in lesions created in underlying scar (59.9 ± 24.2% vs. 35.5 ± 19.7%, p < < 0.01) (Fig. 7e). This difference was likely a result of the reduced wall thickness in these areas of chronic scar (9.0 ± 3.4 mm vs. 14.3 ± 2.0 mm, p < < 0.01).

**Fig. 8:**
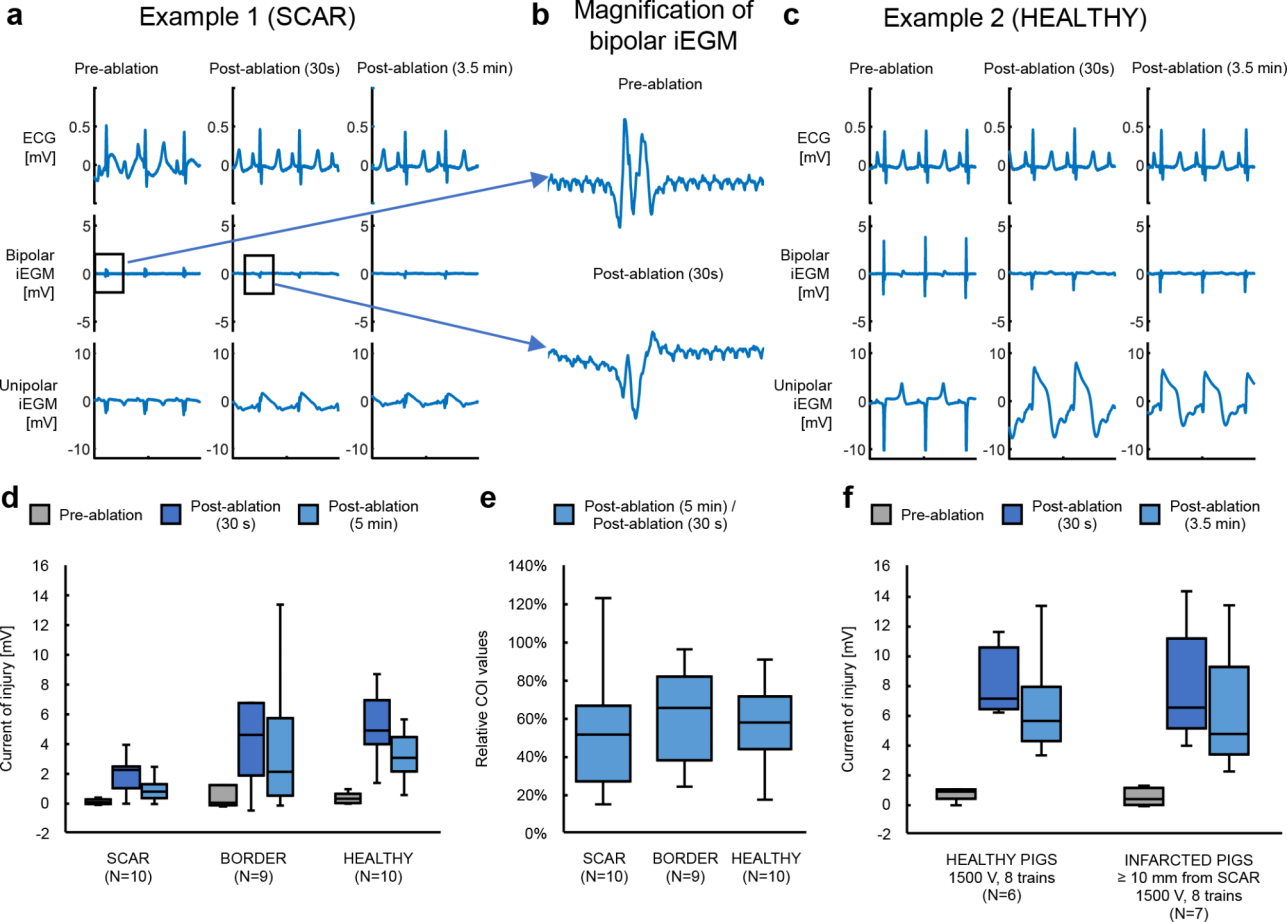
(**a**) and (**c**) Examples of signals recorded at two different ventricular sites in scarred (**a**) and healthy tissue (c; 27 mm from the edge of scarred tissue based on histology) when treated with the same PFA dose (1500 V, 8 trains) in the same animal and shown for three different time points (pre-ablation, 30 s post-ablation and 3.5 min post-ablation). (**b**) Magnified depolarization component of bipolar signals (top: before ablation; bottom: 30 s after ablation). (**d**) Absolute COI measured at three different time points (pre-ablation, 30 s post-ablation and 5 min post-ablation) grouped by the targeted tissue type (scar, border, healthy). An LME model of the form COI ~ Time * Tissue + (1|Pig/Lesion) revealed significant difference between border & scar, averaged over all time points (* *p* = 0.01). (**e**) Normalized COI (5 min post-ablation values normalized to 30 s post-ablation values). No statistically significant differences between the tissue types were observed using a model of the form relative *COI ~ Tissue + (1|PigID)*. (**f**) Comparison of absolute COI values (pre-ablation, 30s post-ablation, and 5 min post-ablation) in healthy myocardium of healthy and infarcted animals. An LME model of the form *COI ~ Time + Pig Model + (1|PigID/Lesion)* revealed significant changes in COI with time post-ablation (*p* < 0.0001 for both post-ablation measurements compared to pre-ablation; *p* = 0.02 comparing 30 s and 5 min post-ablation measurements). No significant difference was detected between healthy and infarcted animals.

Differences in changes observed in iEGMs between scar, border and healthy tissue are shown in Fig. 8. In panels a-c, we compare the responses between lesion sites with the same PFA dose in scarred and healthy tissue of the same animal. In scarred tissue, the baseline peak-to-peak voltage of bipolar iEGMs was much lower than in normal tissue; in the example in Fig. 8a and c (middle row) the difference was more than 7-fold (0.8 mV vs. 5.9 mV respectively). Figure 8b shows a magnification of the depolarization phase of the bipolar iEGM before and after PFA delivery. Fractionation, present before PFA, was reduced to only two dominant peaks after PFA. The increase of COI observed after PFA in unipolar iEGMs was less pronounced in scarred than in normal tissue (Fig. 8d); in the example in Fig. 8a and c (bottom row) the difference was about 2-fold (2.5 mV vs. 6,0 mV respectively). Relative reduction of COI after ablation was similar for scarred and normal tissue (Fig. 8e). We found no significant difference in response to PFA for the heathy myocardium between the healthy pigs and infarcted pigs’ cohorts (Fig. 8f).

LET in the chronically infarcted swine model was determined at 48 h post PFA and compared to LET determined in healthy pigs at 24 h. Lesions located at least 10 mm from the scarred tissue were considered as healthy. Comparable LETs in infarcted and in healthy pigs were found to be 456 V/cm (median value, *N* = 7) and 417 V/cm (median value, *N* = 7), respectively. The difference in LET was not statistically significant (*p* = 0.11, Kruskal-Wallis test).

All summary data used in preparation of Figures is available in the associated repository.

## Discussion

### Overview of clinical implications

Most current PFA systems either approved or in the approval process create lesion depths of 3–5 mm, which are effective for atrial – specifically left atrial – ablation. However, for ventricular ablation, lesion depths of 7–10 mm are often needed. Although ablation of ventricular arrhythmias (VA) is less common than ablation of atrial arrhythmias, VA such as ventricular tachycardia are life-threatening and therefore represent a very important clinical application.

This paper effectively shows how just delivering more pulse trains creates a plateau effect in lesion size. Some have suggested that lesion depth can be increased with greater contact force^35^. There is little doubt that proper contact between the PFA catheter and the tissue is required for optimal lesion formation^30,36,37^. As the catheter moves out of contact from the tissue, lesion width and depth are reduced. The role of contact force, however, is controversial. In the Mattison et al. study, changing contact force created a very small change in lesion depth with a very weak correlation coefficient. Even in studies that suggest that contact force is important^35,38^, it is actually the number of PFA applications and not the contact force which is really driving the lesion depth. In the Di Biase study, changing contact force beyond 5–25 g yielded very little change in lesion depth (with 9X applications, for example, depth increased from 3.25 mm to 3.87 mm at 5–25 g versus 51–80 g respectively).

Another possible way to increase lesion depth is altering the electrode geometry. Utilization of a monopolar delivery catheter with a return catheter in the inferior vena cava as was explored here, along with application of more trains, allows for achievement of lesion depths that may be useful for VA ablation. In normal myocardium, durable lesions were observed by LGE cMRI 6 weeks after PFA and correlated well to the size determined via gross pathology (R^2^ = 0.78, *p* < 0.01, Fig. 3e). In addition, lesion sizes were dose-dependent (Fig. 3a-c). Using numerical modeling and cMRI, we demonstrated the nonthermal nature of PFA even for 16-pulse trains at 1500 V (for the used waveform), which holds the promise of avoiding detrimental side-effects of thermal injury.

The endpoint of most (thermal) ablation procedures is the elimination of bipolar iEGMs. In this study we observed a rapid elimination of the depolarization component of bipolar iEGMs even in cases where no durable lesions were subsequently observed (this replicates what has been seen in other studies^39,40^). However, we have novelly shown that unipolar iEGMs may predict histologically durable lesions created by PFA.

For the first time, we determined the Lethal Electric Field (LET), that is, the threshold for irreversible tissue damage, in vivo in clinically relevant, large animal ventricular tissue. We also demonstrated that LET was similar in the ventricular myocardium of both healthy and chronically infarcted pigs.

Finally, most ventricular ablations are performed in scarred human ventricles. Traditional thermal ablation sources (like radiofrequency energy) have difficulty penetrating through such chronic scar tissue. However, we show in this manuscript that PFA penetrates scar very well and can homogenize regions of varying viability within the scarred tissue and therefore should be able to eliminate critical isthmuses for VA.

### Delineation between thermal and nonthermal origin of lesion

PFA is frequently described as a nonthermal ablation modality, where cell damage is achieved by membrane electroporation^41^, the general belief is that the electric field is not applied for sufficient durations to produce a lethal thermal dose in tissue^42,43^. The nonthermal nature of PFA would support an improved clinical safety profile with less potential for collateral thermal injury than clinically standard thermal modalities such as RFA^44^. However, high PFA doses may cause bubble release^45^which are of thermal origin. Also a small temperature rise (< 8 °C)^2^, in an experimental setting^2^ was reported. We therefore evaluated whether the PFA delivery scheme used in our animal study produced tissue heating sufficient to cause thermal injury.

Two approaches were taken: tissue temperature numerical modelling and in vivo imaging using a temperature-sensitive MRI technique (T1w MRI). Our modelling of the temperature distribution at the PFA site (Fig. 1) estimated minimal heating around the tip of the catheter electrode in contact with myocardium, further mitigated by heat dissipation due to circulating blood. This prediction was supported in the animal experiment by the absence of visible lesions in T1w MRI. At approximately 55–65 °C, oxidation of ferrous (Fe^2+^) to ferric (Fe^3+^) iron within hemoglobin and myoglobin occurs. These met-forms (methemoglobin and metmyoglobin) are paramagnetic molecules and act as endogenous contrast agents^46,47^. While LGE cMRI confirmed the presence of PFA lesions, no enhanced lesions were observed using T1w MRI at these same sites (Fig. 1), supporting our understanding that the PFA injury as performed in our study was largely of nonthermal origin.

### PFA Lesion development dynamics from MRI

Late Gadolinium Enhancement (LGE) cMRI indicated substantial increase of the tissue volume accessible to Gd 24 h post-PFA in tissue exposed to electric field higher than the LET. This observation is consistent with acute inflammation and increased (micro)vascular permeability leading to increased interstitial space. In addition, there is a core contiguous region of PFA-treated cells that underwent cell death, due to increased cell membrane permeabilization, including permeabilization to Gd, resulting in hyperintense signal regions in the cellular compartment of the ablated tissue (see histology in Fig. 4 and Supplemental Figure S1).

Contrary to expectations – given the nonthermal nature of PFA - we observed a hypointense central region in LGE cMRI at 24 h. (e.g., Figs. 2c and 4d) in 7/37 lesions (19%). Such a core tended to be observed at the highest dosing. This appearance is consistent with microvascular obstruction (MVO) leading to hypoperfusion, thus impeding Gd accumulation. While we cannot prove that this dark central region is due to microvascular obstruction, available histopathology of such lesions showed interstitial edema, influx of inflammatory cells, and red cell extravasation at 7 h (Supplemental Figure S1) and 48 h (Fig. 4). MVO or “no reflow” refers to small vessel changes that are frequently observed within the lesion after myocardial infarction^48^, RF ablation^49^, chemoablation^50^, and cryoablation^51^. In the absence of thermal injury, we suggest that the mechanism for MVO in these PFA lesions is similar to MVO during infarction. The development of edema and increased interstitial pressure can compress the microvasculature^52^, reducing the potential inflow of blood and contrast agent relative to normal tissue. Supplemental Figure S1shows an example of inflammation as early as 7 h after PFA in a histological sample. Hemorrhage may also further contribute to the inflammatory response and edema^53^ in the heart. In our study, hemorrhage (extravasated red blood cells) was observed in acute histological samples showing lesions created with the higher PFA doses (e.g., Fig. 4e, showing a 1500 V 8-train lesion). Similar observations were reported by others^54^.

### Electrophysiology of PFA

The surprising lack of change in overall peak-to-peak values of bipolar iEGMs with the number of pulse trains at 1500 V (Fig. 5d and e, right) was also reported by others^54^. The most likely explanation for this is the opposing effect of PFA on reduction (often approaching disappearance) of the depolarization component and a large increase of the current of injury (COI) component immediately after PFA. For unipolar EGMs, the COI was much more pronounced for 16 versus 4 pulse trains, as shown in Fig. 5f for the unipolar iEGM data. Comparable findings were also reported by others^39^, with high-density unipolar epicardial iEGM recordings the authors found more pronounced COI (ST-segment elevation) for signals recorded at the center of the lesion compared to signals reported in the border or periphery.

COI measured in unipolar iEGMs showed a dependence on the number of pulse trains at 1500 V; specifically, the relative COI post-ablation (Fig. 5f right) and its post-ablation recovery (Fig. 5g) increases with increasing number of pulse trains. In contrast, dose-dependence of COI was absent for different voltages when delivered via 4 pulse trains (Fig. 5f and g left). In most cases of low PFA doses, no actual durable lesion or only very small lesions (less than 100 mm^3^ in size) were found in the pathology examinations 6 weeks post-ablation. Two further conclusions can be made. First, the changes in iEGM signals recorded after PFA at the sites where lesions were absent or very small can be attributed primarily to reversibly electroporated (or otherwise temporarily electrically silenced) cells; it is at these low doses where COI from unipolar iEGMs fail to show dose dependence. And second, at higher doses for which lesions were mostly found in gross pathology examinations, the COI from unipolar iEGMs shows a clear dose dependence.

In excitable cell, increased membrane conductivity (manifested as additional nonselective leak current) is able to trigger an action potential, modulate it, and depolarize the cell membrane rendering it transiently unexcitable^55–57^. As the increase of membrane conductivity due to electroporation is rapid but generally transient, this explains both the disappearance of iEGMs immediately following PFA application and their subsequent recovery within a few minutes^56,58^.

Our observations indicate that, in the case of PFA, the unipolar iEGMs recorded over an extended frequency bandwidth contain more valuable information for quantifying the effects of PFA during the procedure than the bipolar iEGMs recorded at standard frequency bandwidth settings. Thus, the COI effect might provide information that could be used (alone or in combination with other parameters) for the development of a treatment index to help guide PFA-based treatment during the procedure. This is also supported by the correlation between the changes in COI from unipolar iEGMs calculated 3.5 min post-ablation and the resultant lesion volumes (Supplemental Figure S2).

### PFA in scarred tissue

One of the most promising aspects of PFA treatment is its ability to penetrate beyond dense scar tissue associated with replacement fibrosis after a myocardial infarction. An important determinant of electric field distribution in heterogeneous tissue is the electrical conductivity of the different tissue layers. For a given voltage delivered via the PFA catheter, the electric field in its locality depends not only on the total impedance of the tissue between the location of the focal/ablation catheter and the location of the return electrodes, but also on the tissue local electrical conductivity. Scar tissue generally has a higher low-frequency conductivity than healthy myocardial tissue because scar is mature fibrosis that is hypocellular but rich in a mature collagenous matrix^16,59^. Therefore, the electric field in scar tissue will generally be lower than in healthy myocardium and scars will not impede the penetration of the electric field; this is shown in Fig. 7c.

### Lethal Electric Field

It is often suggested that cardiac tissue is more sensitive to electroporation than other tissues; that is, it has a lower threshold for damage. However, the lethal electric field threshold (LET) for cardiac tissue in vivo remains undetermined. The median LET in vivo for four trains was 520–562 V/cm when determined from LGE lesion volumes measured 7 days or 6 weeks post PFA. This is comparable to the recently published median LET of 535 V/cm determined for four trains in porcine hearts ex vivo (*N* = 51 lesions in *n*= 6 hearts) even though the time point and assay for determining cell death were different^25^. Figure 6 also shows that LET can be reduced by applying more pulses, but there is diminishing return for applying additional pulse trains.

The concept of LET is appealing and enables prediction of lesion size (particularly depth) even in complex tissue like ventricular myocardium. However, LET depends on pulse parameters and the waveform, as well as when and how (e.g., TTC vs. LGE cMRI) lesion size was determined^25,60^. Our analysis also shows that additional trains have diminishing incremental contributions to the depth of lesion. Since LET depends on the waveform and number of trains^24^, it is important to determine the LET for a specific waveform or at least establish what is the LET difference between different waveforms. It is known that long monophasic pulses (e.g., of 100-µs duration)^25,61^are more efficient in achieving electroporation than short biphasic pulses (e.g. HFIRE). LET as determined in tissue also depends on the numerical model used to calculate electric field as the electric field distribution is compared to lesion size^62^.

Lastly, it is important to note that the electric field during PFA is also present in the blood pool around the catheter (see Fig. 6f) and thus can potentially affect blood cells. It was shown that platelets can be activated by electroporation^63^, that electroporation modifies the activation of neutrophils^64^, and that electroporation leads to hemolysis^65,66^. The volume of blood exposed to electric fields of over 1000–1500 V/cm calculated in the resultssection refers to the volume exposed during a single train of pulses. In reality, the blood would be exchanged between different pulse trains, with the extent of hemolysis depending on the number of pulse applications^67–69^and the catheter design^70^.

## Limitations

The series of studies presented here utilized the same waveform used by the PulseSelect™ PFA system (Medtronic, Inc.). Its waveform is neither optimized for thick-walled ventricular ablations, nor for a bipolar delivery between two catheters. LET was determined for this specific waveform and may have different values when using different waveforms and numbers of trains of pulses. Observations of lesion dynamics via time-based cMRI after PFA were only partially accompanied by contemporaneous histology. Healthy animals were sacrificed at the end of the 6-week follow-up time, which allowed only a comparison of gross pathology and histology to the 6-week cMRI. Animals with pre-existing infarction were sacrificed at 48 h following PFA; this we could make effective comparison between the two g cMRIs acquired at 24 h in the two groups but could not compare gross pathology and histology between the groups as these were done at very different time points. We also did not control the contact force during PFA, but contact force was previously shown to be less impactful than the mere contact to tissue itself^38,54^. LET was determined by comparing the total volume of observed lesions in cMRI with electric fields from numerical modeling; the exact lesion shape was not taken into account. We also did not control the catheter angle relative to the ventricular wall during PFA but estimated via numerical evaluation that the changes in angle and tissue indentation by catheter would result in a variation of about ± 20% in the total lesion volume (Supplemental Figure S4).

## Conclusions

Here, we summarize what we consider to be the most important observations from this study. Lesion size in the porcine left ventricle depends on amplitude and number of Pulsed Field applications. We have been able to achieve 7–10 mm lesion depth as seen in gross pathology with the highest dose used. Numerical modeling and temperature sensitive native T1 MRI imaging confirmed the nonthermal nature of PFA damage with the chosen protocols. LGE cMRI determined lesion size at 7 days and 6 weeks showed good correlation with that measured from gross pathology at 6 weeks. A reduction in LGE-delineated lesion volume from 24 h to 7 days post ablation suggested early resolution of acute injury. Comparison of lesion size from histology with numerically modeled electric field distributions allowed us to determine electric field thresholds for irreversible cardiac tissue damage; these were similar in healthy and infarcted pigs. The delivery of electric pulses resulted in profound morphologic changes in bipolar and unipolar iEGMs. First, there is an immediate reduction of the depolarization component of bipolar iEGM signals, as normally observed after thermal ablation. Second, for unipolar iEGMs we consistently observed a current of injury (COI) phenomenon, which resembles ST segment elevation observed during acute myocardial infarction. COI which was determined from unipolar iEGMs at 3.5 min post-ablation could be used as a potential intraprocedural index of expected lesion extent since it was correlated to lesion sizes measured from gross pathology (correlation R^2^ = 0.77). Conversely, no correlation between the reduction of the bipolar iEGMs and lesion size was observed. The results obtained in the infarcted pig model suggest that ischemic scar does not impede PFA lesion depth. We show that we achieve average depths of 4–5 mm in both healthy and infarcted tissue with 8 trains at 1500 V. The capacity to typically achieve about 60% transmurality in infarcted tissue (due to effective scar penetration and thinner walls in this area) is also encouraging in the context of future use in managing ventricular arrhythmias.

## Methods

The study is reported in accordance with ARRIVE guidelines (https://arriveguidelines.org).

### Animal model and ethical approval

All animal work was performed at Sunnybrook Research Institute (SRI, Toronto, Ontario, Canada), within the Department of Preclinical/Comparative Research. This facility operates under the regulations of the Canadian Council for Animal Care (CCAC) with a “Certificate of GAP-Good Animal Practice” in good standing. This study protocol was approved by the Sunnybrook Research Institute (SRI) Institutional Animal Care and Use Committee (IACUC). All methods in this animal study were performed according to the CCAC guidelines for GAP, and as ethically approved by the SRI IACUC.

Ten healthy and 5 infarcted animals were included in the study. Yorkshire cross-bred swine, both neutered males and intact females, weighing 20–30 kg on arrival were procured from Lifetime Solutions (Newcastle, Ontario, Canada), and transferred to the animal facility a minimum of 7 days for acclimation prior to any procedure. They were housed individually on a 12-hour light cycle, with ad lib access to water, and fed LABDIET™ Porcine Lab Grower diet.

### Preoperative procedure

Pigs were fasted for 12 h prior to all general anesthesia (GA) procedures with ad lib access to water. They were sedated with an intramuscular (IM) injection of ketamine at dose of 15–33 mg/kg (Narketan 100 mg/ml), followed by O_2_ (2 L/min) and isoflurane (1–5%) via facemask for anaesthetic induction. A 22G angiocath was inserted into the marginal ear vein for intravenous (IV) access and intraoperative maintenance fluids (NaCl 0.9% 3–5 mg/kg/hr). The animals were intubated with an ET tube (7.0–8.0 F) and mechanically ventilated with O_2_ (2 L/min) and isoflurane (1–5%) with a tidal volume of 10–15 mL/kg to maintain a surgical plane of anesthesia. Blood oxygen saturation (SPO2), end-tidal carbon dioxide (ETCO2), ECG and body temperature were monitored during all GA procedures and all animals received an IM injection of ceftiofur antibiotic and a subcutaneous injection (SQ) of slow-release, long-acting buprenorphine 0.02–0.12 mg/kg (Chiron Compounding Pharmacy, Slow Release Buprenorphine 10 mg/mL) for analgesia (effective for 36–48 h) prior to surgery. Twice-daily assessment of animals occurred post-operatively.

### Myocardial infarct (MI) procedure

The left femoral artery was visualized using an ultrasound linear array probe (Siemens Sequoia) and cannulated with a 6 F sheath using the Seldinger technique. After sheath placement, the animal received an IV bolus of heparin at 100 IU/kg for anticoagulation (heparin sodium injection 1000 IU/mL) and 50 mg of beta-blocker (amiodarone hydrochloride 50 mg/mL) to help manage procedurally related arrythmias. A guiding catheter (Launcher JR-3.0, Medtronic, Minneapolis, MN, USA) was advanced to the coronary ostia under fluoroscopic guidance and an angiogram of the coronary vessels was recorded for reference using injection of 10–15 cc contrast dye (Omnipaque 350, 755 mg of iohexol/mL). An angioplasty balloon (Medtronic Sprinter Legend) was placed within the left anterior descending (LAD) coronary artery, positioned just distal to the 1st LAD diagonal branch. The balloon was inflated (Bard Eagle 10 cc/30 atm) to occlude blood flow. After 100 min of occlusion the balloon was deflated and removed to allow for LAD reperfusion, as confirmed by angiogram. The pig recovered and returned to its pen. Four to six weeks after MI, it underwent PFA.

### Pulsed field ablation - cardiac access

Healthy or chronically infarcted pigs were sedated and intubated following the same preoperative protocol. Using ultrasound guidance, a 9 F sheath was placed within the right femoral artery for a retrograde aortic approach, and 6 F and 7 F sheaths were placed in the left femoral vein. Once access was gained, the animal was heparinized (100 UI/kg). A 6 F decapolar catheter (Inquiry™ Abbott Medical, Plymouth, MN, USA) was positioned in the coronary sinus as a reference and a 7 F decapolar catheter (Marinr™ Medtronic) was positioned in the inferior vena cava (IVC) as a return electrode. Before delivery of PFA the animal was given an IV bolus of rocuronium 0.6–1.2 mg/kg (rocuronium bromide 10 mg/mL) to mitigate skeletal muscle contraction. A high density electroanatomical map (EAM) of the left ventricle (LV) was created using a multipolar mapping catheter (Advisor™ HD Grid Abbott Medical, Plymouth, MN, USA) and the EnSite Precision™ Mapping System (Abbott, Plymouth, MN, USA). After the EAM was completed, the focal PFA catheter was advanced to the left ventricle (LV). Five to six ablations per animal were delivered in different segments of LV (basal, mid, apical, and anterior, septal, lateral, posterior) with enough separation to allow individual analysis. A Medtronic research-only PFA generator was used, with PFA parameters derived from clinical use and optimized for atrial deliveries. In the infarcted cohort of animals, lesions were delivered in dense scar, border zone/heterogeneous scar, and healthy myocardium.

### Pulsed field ablation - PFA treatment

Pulsed Field Ablation was delivered from a tip electrode of an 8 F, 5 mm tip focal catheter (Conductr™, Medtronic, Minneapolis, MN, USA) to all electrodes on a decapolar CS catheter (Marinr™, Medtronic, Minneapolis, MN, USA). The waveform consists of a biphasic pulse train lasting approximately 100 ms. Energy was delivered in a voltage-controlled manner ranging from 1000 V to 1500 V, with all other waveform parameters being held constant. The number of pulse trains delivered was 1, 4, 8 or 16, with each train requiring about two seconds for delivery. Energy delivery was timed to the R wave using an external cardiac trigger monitor (IVY 7600EP, IVY Biomedical, USA). Measurements of peak current during each pulse train were recorded by the firmware and used for development and validation of the numerical models.

### Cardiac MR imaging

MR imaging was performed using a 3 Tesla scanner (MR750, GE Healthcare, Waukesha, Wisconsin) with an 8-channel anterior cardiac coil. Imaging was repeated at 24 h, 1 week, and 6 weeks post-PFA. Before and during the scan, animals were intubated and anesthetized with continuous isoflurane (see Preoperative Procedure). Native T1-weighted MRI was performed using a prototype 3D inversion recovery prepared spoiled gradient echo sequence with a B_0_- and B_1_-robust inversion pulse. Diaphragmatic navigation was used for respiratory gating; native T1-weighted imaging time for whole-heart coverage ranged between 10 and 15 min with > 50% respiratory navigation efficiency. Nominal sequence parameters include: TI = 700–800 ms; resolution = 1.4 × 1.4 × 2.6 mm^3^; TR/TE = 3.5/1.5 ms, flip angle = 15°; readout bandwidth = 100 kHz; and a recovery period between preparations of 4 R-R intervals (TI is inversion time, TR is repetition time, and TE is echo time). LGE cMRI was performed 15 min after a bolus injection of gadobutrol (0.2 mmol/kg, Gadavist, Bayer Healthcare Pharmaceuticals, Berlin, Germany). Sequence parameters were identical to the native T1-weighted MRI, with the exception of resolution = 1.4 × 1.4 × 1.4 mm^3^ and TI = 300 ms. LGE cMRI acquisition duration ranged between 8 and 10 min with > 60% respiratory navigation efficiency.

### MRI-derived scar maps

The pre-ablation 3D LGE cMRI was used to create an MRI scar map for each infarcted animal. Pixel signal intensity thresholds of 40% and 60% relative to maximum values across the LV myocardium were used to differentiate healthy tissue (< 40%), heterogeneous scar (40–60%), and dense scar (> 60%) (ADAS 3D, Barcelona, Spain). Furthermore, scar maps were determined in 5%-layer increments from the subendocardial to the subepicardial layers to characterize scar transmurality. These scar maps were used to identify locations to prescribe focal lesions in scarred and healthy regions. Anatomical landmarks including the right coronary ostium, left coronary ostium, LV apex, and inferior and superior deflections of the aortic arch, were manually segmented from the LGE scar maps and tagged in the bipolar voltage maps. Identification and manual alignment of anatomical landmarks in MRI scar maps and bipolar voltage maps facilitated the post-ablation follow-up MRI assessment of ablation lesions according to ablation markers provided by the EP navigation system (EnSite Precision, Abbott).

### MRI and gross pathology - lesion volume, depth, and width measurements

Lesion volumes were measured using 3D LGE cMRI 15 min post-Gd injection acquired at 24–48 h, 7 days, and 6 weeks post-ablation. Focal lesion volume was manually segmented (ADAS 3D, Barcelona Spain) as hyperintense regions within the myocardium. Focal lesion depth was measured starting from the endocardial border to the maximum extent of the lesion in short axis orientation and lesion width was measured as the maximum straight-line distance of the lesion through the myocardium in short-axis orientation using 3D LGE cMRI 15 min post-Gd injection. At the 6-week time point, the pigs were sacrificed under GA by IV injection of 20 cc of a supersaturated potassium chloride (KCl). After death was confirmed, the heart was explanted and immersion-fixed in ample 10% formalin for a minimum of 3 weeks. The whole hearts were prepared for gross pathological slicing by resecting the atrial appendages and embedding the ventricles in Cavex ColorChange alginate gel (Cavex BV, Haarlem, Netherlands). The embedded heart was then sliced from apex to base in a short-axis orientation in 2 mm increments and visually examined for lesions. Lesion width and depth measurements were made from digital images that were acquired using a focal camera with a fixed field-of-view and source-to-object distance to ensure consistent image magnification. Trajectories of the depth and width measurements in the fixed tissue slices were similar to those from the 3D LGE cMRI method (Fiji, Image J). The correlation between lesion width and depth measurements using gross pathology slices and 3D LGE cMR images was evaluated. For the chronically infarcted hearts, the acute PFA lesions that were either fully or partly in healthy or in scarred tissue were similarly assessed. If an ablation was ≥ 10 mm from scar tissue, it was categorized as “healthy”. Typically, lesion measurements in infarcted hearts are based on LGE cMRI acquired 24–48 h post-ablation. Lesions were excluded from analysis when not identifiable in gross pathology or when there was uncertainty as to the corresponding PFA delivery.

### Wall thickness measurements

3D LV shells were constructed using the 24-hour 3D LGE MR images (ADAS 3D, Barcelona, Spain). Bipolar voltage maps were used for focal lesion localization and aligned to LGE cMR images using rigid registration. The right coronary ostium, left coronary ostium, superior and inferior deflection of the aortic arch, and the LV apex were used as registration landmarks between the bipolar voltage maps and the endocardial layer of the LV shell. LV wall thickness was measured from MRI (Fig. 4a) using short-axis images from the reformatted 3D LGE cMR image volumes. Consistent image orientations were ensured across all animals by orienting one long-axis localizer transecting the LV apex to the aortic root and another long-axis localizer transecting the LV apex to the mitral valve. The short-axis image orientation was then perpendicular to these long-axis localizers. Wall thickness was determined by measuring the largest straight-line distance from the endocardium to epicardium at the greatest depth of each PFA lesion visualized in 3D LGE cMR images in short-axis orientation (ADAS 3D, Barcelona, Spain).

### Catheter location reconstruction

The intracardiac positions of the three catheters (PFA, return, electrophysiology) was reconstructed from the fluoroscopy images. For each ablation point, a fluoroscopy image series comprising at least one heartbeat was acquired at two angles that were ≥ 45˚ apart. The image closest to the diastolic state of the heart was identified in each series and the three catheters were manually traced using a custom graphical user interface in Matlab (Mathworks Inc, Natick, MA, USA). A cubic spline was fitted to the catheter tracings. The cubic spline was interpolated in 1-mm increments to advance the pixel position on the primary image. On the second image of each ablation acquisition, a corresponding location on the tracked catheter was identified by finding the location on the spline with identical y-coordinate. The pair of points was then used to reconstruct a 3D position for the intersection of the two beams on the line between the X-ray source and the pixel on the detector by considering the distance between the source and detector stored in the DICOM format (Supplemental figure S5a). This was repeated for the entire catheter trajectory for all catheters in each image. In this way, the trajectories in 3D space were determined for the three catheters used in each treatment location. The distance between the object and the detector was determined by measuring the length of the reconstructed return catheter (which was always straight and located in the IVC) and the length of the catheter in the fluoroscopy image. The length in the fluoroscopy image could be accurately calibrated using the known distances and dimensions of the 10 electrodes on the Marinr™ catheter.

We generated a geometric 3D representation from the catheter trajectories and exported it to 3-matic (Materialise, Leuven, Belgium). There, the ensemble of all catheters used in each treatment (ablation, return and CS at 5–6 different sites) was aligned to the 3D reconstruction of the heart (Supplemental figure S5b) by aligning the catheters to the different landmarks of the MR images (CS, IVC, aorta, apex) (Supplemental figure S5c). Similar methods were used previously^71,72^. While Wagner et al.^72^ achieved localization accuracy of below 1 mm, our study showed a localization RMSE of the ablation tip of ~ 9 mm; however, Wagner et al. had an immovable geometry in the cadaver and phantom studies, while the catheters in our study were moving with the heartbeat and respiration.

### Numerical modeling

The numerical modeling in this study consisted of several different numerical models constructed for different purposes. The model used for LET determination was a schematic model with an idealized geometry. We used a more detailed animal-specific model for modeling ablation in infarcted ventricles. Both of these models used a stationary study to determine the electric field distribution in tissue. Finally, a time-dependent study was used for the schematic model to determine the heating in tissue under different blood flow conditions to determine the possible extent of thermal damage in tissue.

### Numerical modeling – electric field and Lethal Electric Field threshold (LET)

A numerical model consisting of the ventricles complex, inferior vena cava (IVC) and the surrounding bulk tissue (Fig. 6d and Supplemental Figure S6a) was built in COMSOL Multiphysics (Version 6.1, COMSOL AB, Stockholm, Sweden). An idealized representation/geometry of the ventricles was constructed using two ellipsoids truncated at the basal plane while neglecting the papillary muscles and trabeculations as described in^73^. For the IVC a simple cylinder was used. The dimensions of the schematic biventricular model and the position of the IVC and the ventricles were determined by scaling and aligning the biventricular model with the measurements of heart size and orientation on the MRI. Geometries of ablation and return catheter were built in Fusion 360 (Autodesk, San Francisco, CA, USA) and imported into COMSOL. The return catheter was positioned in the IVC based on the catheter location reconstruction (see above). The ablation catheter was positioned in the middle of each of the 13 myocardial segments shown in Fig. 6e, perpendicular to the myocardium wall with a 0.5-mm indentation into the tissue (Supplemental Figure S6b).

The myocardium was modeled as an anisotropic material with three orthogonal directions: Fiber direction, sheet direction, and normal to sheet direction (Supplemental Fig. 6c). For the sheet direction, the transmural direction was used, and for the fiber direction, a linear variation of the fiber angle across the wall was assumed with a fiber inclination of 80° at the endocardium and − 40° at the epicardium^74,75^. This reference system, which was used to describe electrical tissue properties, was created in two steps. First, the transmural direction was obtained using the Curvilinear Coordinate interface and the diffusion method, which solves the Laplace equation (Eq. 1) for the specified inlet ($\:U=1$ at the endocardium boundary surface) and outlet ($\:U=0$ at the epicardium boundary surface) boundary conditions.

1 $$\:{\Delta\:}U=0$$

The gradient of the solution $\:U$ forms the first basis vector and is oriented in the sheet direction. The second basis vector was defined manually in the apico-basal direction and the third basis vector was the cross product of these two. The resulting curvilinear system is then rotated with an angle $\:{\Theta\:}$ around the first basis vector to align the third basis vector along the fiber direction, as described in Eq. (2).

2 $$\:{\Theta\:}={\upbeta\:}{\cdot\:{\Theta\:}}_{\text{e}\text{n}\text{d}\text{o}}+\left(1-\beta\:\right)\cdot\:{{\Theta\:}}_{epi}$$

Here $\:{{\Theta\:}}_{\text{e}\text{n}\text{d}\text{o}}$and $\:{{\Theta\:}}_{epi}$ represent fiber inclination at the endocardium (80°) and epicardium (−40°) boundary respectively, and $\:{\upbeta\:}$ is a dimensionless parameter representing the distance to the epicardium boundary, as described in Eq. (3). It takes values between 0 (epicardium) and 1 (endocardium).

3 $$\:\beta\:=\frac{{D}_{epi}}{{D}_{epi}+{D}_{endo}},$$

where $\:{D}_{epi}$ and $\:{D}_{endo}$ represent the fiber distance to the epicardium and endocardium and were calculated using the Wall distance interface in COMSOL.

To model the electric field in myocardium, the Electric Currents interface was used in COMSOL, where the continuity Eq. (4) for conservation of charge was solved for steady state electric fields in a stationary study:

4 $$\:\nabla\:\cdot\:(\sigma\:\nabla\:V)=0,$$

where $\:V$ is the electric potential and $\:\sigma\:$ is the electrical conductivity. An electric potential boundary condition was applied at the electrodes of the return and ablation catheter. Voltage at the electrodes of the return catheter was set to zero and voltage at the ablation catheter electrode was set to the delivered voltage (1000 V, 1300–1500 V). At all other boundaries, electric insulation (i.e., zero normal electric current through the boundary) was specified as a boundary condition. A 3% voltage drop at the ablation catheter electrode was considered to account for voltage loss in the catheter internal wire resistance.

Electrical conductivity of myocardium ($\:{\sigma\:}_{myo}$) was modelled as anisotropic and electric field dependent, as described in Eq. (5).

5 $$\:{{\sigma\:}_{myo}\left(E\right)=\sigma\:}_{\begin{array}{c}\parallel\:,\:\perp\:\\\:\:\end{array}}\cdot\:{f}_{E}\left(\left|E\right|\right).$$

Electrical conductivity along the myocardial fibers ($\:{\sigma\:}_{\left|\right|}=0.5\:S/m$) as well as the anisotropy ratio ($\:AR=\frac{{\sigma\:}_{\parallel\:}}{{\sigma\:}_{\perp\:}}=1.34$), were taken from^25^. Electrical conductivity perpendicular to the myocardial fibers ($\:{\sigma\:}_{\perp\:}$) was calculated as $\:{\sigma\:}_{\perp\:}=\frac{{\sigma\:}_{\parallel\:}}{AR}=0.375\:S/m\:$. Change in electrical conductivity due to electroporation was modelled with a smoothed Heaviside function with 2 continuous derivatives. Specifically, the function $\:{f}_{E}$ represents a smooth increase from 1 to the electroporation conductivity increase factor ($\:EF\:=\:2.65)$, with transition region fixed at 550 V/cm and width of the transition region 500 V/cm^25^. Electric conductivity of blood ($\:{\sigma\:}_{blood}=0.7\:\text{S}/\text{m})$ was taken from IT’IS database (blood, $\:10\:\text{k}\text{H}\text{z}$). Electrical conductivity of bulk tissue ($\:{\sigma\:}_{bulk}=0.38\:S/m$) was adjusted to align the values of calculated electrical current in the model with experimentally measured values. All material properties used in the numerical models and their references can be found in Supplemental Table S1.

Lethal electric field thresholds were calculated as follows: (i) we assigned a myocardial segment to each in vivo PFA lesion based on the 13 segment model (Supplemental Fig. 6b); (ii) we used the bisection method to find the electric field threshold LET_i_ that results in the same volume of myocardium exposed to electric field above the LET_i_.

6 $$\:{V}_{model}=\iiint\:E\ge\:{LET}_{i}\:dV$$

Equation (6) shows the integration used to obtain the tissue volume which was compared with the volume of the MRI lesions obtained at different time points post ablation (24 h, 1 week, 6 weeks), iii) we calculated median LET for all lesions that received the same PFA dose for each imaging time point post ablation.

### Numerical modeling – PFA in scarred tissue

To model ablation through scar (Fig. 7c), the model geometry consisted of the exact LV pre-ablation shell exported from ADAS and a simple cylinder representing the IVC which was positioned in relation to the LVs based on the MRI reconstructions. The ablation catheter was positioned at the lesion location determined from the electroanatomical map ablation tags and the return catheter was positioned in the IVC based on the catheter location reconstruction for the same lesion. The three tissue types were included in the model by importing the scar maps from ADAS (determined from LGE cMRI as described above) into the model as scar function. The scar function ($\:{f}_{scar}$) prescribed values 0 to healthy myocardium (< 40% pixel signal intensity), values of 1 to dense scar (> 60% pixel signal intensity) and values between 0 and 1 were used to describe heterogeneous scar (40–60% pixel signal intensity).

Electrical conductivity of healthy myocardium was modeled as anisotropic and electric field dependent, as per Eq. (5) - $\:{\sigma\:}_{myo}\left(E\right)$. Electrical conductivity of scar tissue was considered isotropic and independent of electric field; a value of $\:{\sigma\:}_{scar}=1\:S/m$ was chosen based on the measurements reported in (Cinca, 1998; Salazar et al., 2004). For the conductivity of heterogeneous scar a linear combination of electrical conductivities of scar ($\:{\sigma\:}_{scar}$) and healthy myocardium ($\:{\sigma\:}_{myo}\left(E\right)$) was used. Equation (7) shows the prescribed electrical conductivity of tissue in the left ventricle based on the scar function.

7 $$\:{\sigma\:}_{tissue}={f}_{scar}\cdot\:{\sigma\:}_{scar}+\left(1-{f}_{scar}\right)\cdot\:{\sigma\:}_{\begin{array}{c}\parallel\:,\:\perp\:\\\:\:\end{array}}\cdot\:{f}_{E}$$

All material properties used in the numerical models and their literature sources can be found in Supplementary Table S1.

### Numerical modeling – temperature and thermal damage

Temperature increase during PFA depends on the interplay of ohmic heating of the tissue and blood during the application of the pulse train and heat transfer through conduction and especially through convection due to blood flow.

To model temperature increase during PFA, Heat Transfer in Solids and Fluids interface was used in COMSOL, where the heat Eq. (8) for conservation of energy was solved in a coupled time dependent study:

8 $$\:\rho\:\text{C}\left(\frac{\partial\:T}{\partial\:t}+\varvec{u}\cdot\:\nabla\:\text{T}\right)-\nabla\:\cdot\:\left(k\nabla\:T\right)-Q=0,$$

where ρ is the density, $\:C$ the heat capacity at constant pressure, $\:k$ is the thermal conductivity, $\:\varvec{u}$ is the fluid velocity field and $\:Q$ is the heat source.

The heat source ($\:Q$) was described as follows:

9 $$\:Q=\text{O}\text{N}\text{O}\text{F}\text{F}\cdot\:\text{d}\text{u}\text{t}\text{y}\cdot\:{Q}_{ec}=\text{O}\text{N}\text{O}\text{F}\text{F}\cdot\:\text{d}\text{u}\text{t}\text{y}\cdot\:\sigma\:{E}^{2}.$$

Ohmic heating ($\:{Q}_{ec}$) is proportional to electrical conductivity ($\:\sigma\:$) and the square of electric field distribution ($\:\varvec{E}$) and is active only during the actual pulse duration during the application of each pulse train. Electric field distribution ($\:\varvec{E}$) was obtained by solving the charge conservation Eq. (4) in the same time-dependent study (bidirectionally coupled). When solving the charge conservation equation, a constant (instead of time-dependent) electric potential boundary condition was applied to the ablation electrode to reduce computational complexity. To then relate the steady ohmic heating source to a pulsating one, first the ohmic heating source was multiplied with the duty factor of the PFA waveform ($\:duty$) to accurately model the heating during the pulse train. To accurately model multiple pulse trains and pauses between the trains, the heating source was multiplied with the $\:ONOFF$ variable, which was switched $\:ON$ at the start of the pulse train and $\:OFF$ at the end of the pulse train. This was done using the Events interface in COMSOL, which forces the solver to take additional time steps and reinitialize the dependent variables ($\:ONOFF$ and $\:T$) at the specified time of the event (start and end of the pulse train).

To account for the cooling of the myocardium, ablation and return electrodes through blood flow, the convection part of the heat Eq. (8), a steady fluid flow in the laminar regime was modeled in the LV and IVC. To model the blood flow in the laminar regime, Laminar flow interface was used in COMSOL, where the incompressible form of the continuity Eq. (10) for conservation of mass and the Navier–Stokes Eq. (11) for conservation of momentum were solved in a separate stationary study, and the solution (blood velocity field $\:\varvec{u}$) was imported into the coupled time-dependent study.

10 $$\:\nabla\:\left(\rho\:\varvec{u}\right)=0$$

11 $$\:\rho\:\left(\frac{\partial\:\varvec{u}}{\partial\:t}+\varvec{u}\cdot\:\nabla\:\varvec{u}\:\right)=-\nabla\:p+\nabla\:\cdot\:(\mu\:\left(\nabla\:\varvec{u}+{\left(\nabla\:\varvec{u}\right)}^{T}\right),$$

where ***u*** is the fluid velocity field, *p* is the fluid pressure, *ρ* is the fluid density, and *µ* is the fluid dynamic viscosity ($\:0.0035\frac{kg}{ms}$was used for blood)^76^.

For the blood flow in the left ventricle, an inlet boundary condition with average flow velocity and fully developed flow (i.e. parabolic laminar flow profile) was specified at the base of the ventricle and an outlet boundary condition of zero pressure was specified near the apex. At the ventricle walls and ablation catheter surface, a non-slip boundary condition was specified. Two values of the average flow velocities and the inlet were chosen, one accounting for the low blood flow scenario ($\:{u}_{low}=3\frac{cm}{s}$) and one for the high blood flow scenario ($\:{u}_{low}=9\frac{cm}{s}$). The inlet velocities were multiplied by the ratio of cross section area at the position of the ablation catheter in the left ventricle divided by the surface area of the inlet, to ensure the average flow velocities around the ablation catheter and the ablated myocardium were 3 cm/s and 9 cm/s for low blood flow and high blood flow scenario respectively, which is similar to that employed in^77^.

For the blood flow in the IVC an inlet boundary condition with constant flow rate and fully developed flow (i.e. parabolic laminar flow profile) was specified at the distal cross section of the IVC. The outlet boundary condition of zero pressure was specified at the proximal cross section at the IVC ostium in the RV. On the IVC inner walls and return catheter surface a non-slip boundary condition was specified. Two values of inlet flow rates were chosen: one accounting for the low blood flow scenario ($\:{F}_{low}=15\frac{ml}{s}$) and one for the high blood flow scenario ($\:{F}_{high}=37\frac{ml}{s}$), with the values taken from^78^. The return catheter was pressed against the IVC wall to model the worst-case scenario.

The following boundary conditions were chosen for the heat Eq. (8). At each blood flow inlet (IVC, LV), an inflow boundary condition was chosen and upstream temperature of 37 °C was specified. On the outer surface of the model, a thermal insulation boundary condition was used (no heat flux across the boundary). The initial temperature of the whole model was set to 37 °C.

The thermal properties of cardiac tissue were modeled as isotropic. The values of thermal capacity ($\:{C}_{myo}=3686\frac{J}{KgK}$) and density ($\:{\rho\:}_{myo}=1081\frac{kg}{{m}^{3}}$) for myocardium were taken from IT’IS database (heart muscle)^79^. Thermal conductivity of myocardium ($\:{k}_{myo}\left(T\right)$) was modeled as temperature dependent, as described in Eq. (12):

12 $$\:{k}_{myo}\left(T\right)={k}_{0}(1+0.0012\left(T-37\:^\circ\:C\right)).$$

Thermal conductivity of myocardium at 37 °C ($\:{k}_{0}=0.56\frac{W}{mK}$) was taken from IT’IS database (heart muscle)^79^. Temperature dependence of thermal conductivity of myocardium was described as linear with the rate of increase of 0.12%^80^.

Electrical conductivity of myocardium was modelled as anisotropic and electric field and temperature dependent, as described in Eq. (13):

13 $$\:{{\sigma\:}_{myo}\left(E,T\right)=\sigma\:}_{\begin{array}{c}\parallel\:,\:\perp\:\\\:\:\end{array}}\cdot\:{f}_{E}\cdot\:\text{exp}\left(0.015\right(T-37\:^\circ\:C)).$$

Temperature dependence of electrical conductivity of myocardium was described as exponential with the rate of increase of 1.5%.^80^

For blood, the values of thermal conductivity ($\:{k}_{blood}=0.52\frac{W}{mK}$), thermal capacity ($\:{C}_{blood}=3617\frac{J}{KgK}$) and density ($\:{\rho\:}_{blood}=1050\frac{kg}{{m}^{3}}$) were taken from IT’IS database (blood)^79^. For bulk tissue, the values of thermal conductivity ($\:{k}_{bulk}=0.39\frac{W}{mK}$), thermal capacity ($\:{C}_{bulk}=2372\frac{J}{KgK}$) and density ($\:{\rho\:}_{bulk}=1027\frac{kg}{{m}^{3}}$) were taken from IT’IS database (connective tissue)^79^. The same electrical conductivities of blood and bulk tissue were used as described in the *Numerical modeling – Electric field and Lethal Electric Field Threshold (LET)* section. All material properties used in the numerical models and their references can be found in Supplementary Table S1.

Compared to RFA, the heating from PFA is not constant but it happens in short bursts during the delivery of pulse trains. For this reason, using the 50 °C isotherm, which is often used in RFA^81^, to evaluate the extent of thermal damage would not give us accurate/realistic results. To account for the pulsatile nature of the heating from PFA delivery, we used the following two methods to calculate the thermal damage. First, we used the “thermal dose” method, where we evaluated the volume of cardiac tissue where the temperature reached 55 °C or more for the damage time of 1 s or more. Second, we used the Arrhenius integral, Eq. 14, to estimate the thermal damage:

14 $$\:{\Omega\:}\left(t\right)={\int\:}_{o}^{t}A{e}^{-\frac{{\Delta\:}E}{RT\left(t\right)}}dt\:,\:\:$$

$\:\text{w}\text{h}\text{e}\text{r}\text{e}\:A$ is the frequency factor, $\:{\Delta\:}E$ is the activation energy, $\:R$ the gas constant and $\:T$ the temperature. The volume of thermal damage was evaluated as the volume of cardiac tissue with a probability of cell death exceeding 63% ($\:{\Omega\:}=1$). The values of $\:A=2.94\cdot\:{10}^{39}\:{s}^{-1}$ and $\:{\Delta\:}E=2.596\cdot\:{10}^{5}\frac{J}{mol}$were taken from^82^.

To model temperature increase during PFA, the workflow of simulations was as follows. First, blood flow was solved in a stationary study, and the solution from the flow study (blood flow velocity distribution) was then used in the subsequent time-dependent study where the temperature and electric field were solved simultaneously by coupling the heat Eq. (8) with charge conservation Eq. (4). The time dependent study was solved for the 16-train PFA protocol. Pauses between trains were set based on the data from the electrical current measurements − 2.5 s between every train and 5 s every 4 trains. In the time-dependent study, timesteps of 0.25 s were specified with one additional time step before and after the application of each pulse train.

### Intracardiac Electrogram (iEGM) measurements

For electrophysiological signal acquisition a clinical recording system (Cardiolab, GE Healthcare) was used, and signals were digitized with a 1-kHz sampling frequency. In addition to standard bipolar iEGMs with typical clinical filter settings (band pass 30–500 Hz), unipolar iEGMs with a broadened frequency range (band pass: 0.5–500 Hz) were recorded together with a standard 12-lead ECG. At each targeted site, iEGMs were recorded for 30 s prior to PFA delivery to establish stable baseline pre-treatment values. The catheter was kept in place manually and the recording continued for up to 5 min after PFA delivery. A 12-lead ECG recording was visually examined and the lead best suited for QRS complex detection was selected. The So-Chan QRS detection algorithm was used and implemented in Matlab^83^. To improve QRS detection, ECG signals were pre-filtered using a high-pass filter with cut-off frequency of 10 Hz, and the first 50 samples were set to zero to avoid the cases in which the segment started with a QRS, which would otherwise prevent the So-Chan algorithm from detecting the next valid QRS complex in the sequence. The better-known Pan-Tompkins algorithm for QRS detection was also tried, but the So-Chan algorithm gave better detection accuracy, especially for low amplitude ECG signals. Correctly detected QRS complexes in ECG were used as fiducial points in subsequent processing of iEGM signals.

All signals were recorded continuously for 5 min after ablation; however, iEGM signals for a significant number of lesions (6 out of 70, both cohorts: in healthy hearts we recorded 40 and included 35 in the analysis; for chronically infarcted hearts we recorded 30 and included 29) were interrupted by obvious movement artefacts before the end of the observation period. Therefore, we limited further processing and analysis only to signals with at least 3.5 min of artefact-free post ablation duration. Unipolar and bipolar iEGMs were processed offline using custom-made code in Matlab R2021b (Mathworks Inc., Natick, MA, USA). The recordings were divided into nonoverlapping 10-s segments and inspected for signal abnormalities (caused by output amplifier saturation, poor electrical contact, lost current return pathway, etc.). Segments with more than 20% of the heartbeats contaminated with such artefacts were excluded from further analysis. Signals were analysed for a period before the delivery of PFA to establish the so-called baseline or pre-treatment values and for a continuous period of 3.5 min following the treatment. Note that delivery of PFA always induced a temporary saturation at the output of the signal amplifiers, most prominently in the case of unipolar iEGMs (an effect analogous to saturation of ECG amplifiers after delivery of defibrillation pulses). In our case, it took up to 30 s after delivery of electric pulses for the unipolar signals to be valid again. For this reason, the post-treatment values of iEGM signals are shown only from 30 s after the delivery of the energy onwards.

For bipolar iEGMs, a detection window of 400-ms duration was created for every valid QRS complex detected in ECG, starting 200 ms before the detected QRS peak, within which the peak-to-peak amplitude of the raw unprocessed bipolar iEGM signal was measured. This parameter was used to monitor changes in bipolar iEGMs. It should be noted that by limiting ourselves to only peak-to-peak measurements in bipolar iEGMs we mimicked the performance of automatic algorithms implemented in voltage mapping systems typically used for acute assessment of iEGMs after thermal ablation in electrophysiological procedures.

In case of unipolar iEGMs, the appearance of a large current of injury component (COI) was by far the most obvious change after application of PFA. Measurement of this effect was based on AUC (area under curve) estimation for the ST segment of each individual heartbeat. The baseline for the COI measurement in unipolar iEGMs was taken from the isoelectric line preceding the QRS for at least 50 ms, and the area under curve (AUC) was calculated in a window starting after the QRS and ending before the start of the T wave. To compensate for uncertainty in the estimation of the start of the T wave (and consequently in variability in ST segment lengths and therefore in AUC), the COI (expressed in mV) was calculated by dividing the AUC by the corresponding ST segment length (see illustration in Fig. 5b). This parameter was used to monitor changes in COI in unipolar iEGMs.

The parameters for bipolar and unipolar iEGMs were therefore calculated for every valid and normal QRS complex from the ECG. For each 10-second-long segment from the original signals with at least 10 valid and normal QRS complexes, the median values of the peak-to-peak amplitudes (for bipolar iEGMs) and of COI (for unipolar iEGMs) were calculated and considered as the representative average amplitudes for the given 10 s segment. This enabled us to follow the changes in these parameters after application of PFA. In presentation of our results, we use the values before the treatment and 30 s and 3.5 min after the treatment.

The discrepancy between the number of bipolar and unipolar iEGMs analyzed and included in Fig. 5 (30 vs. 35), is due to a protocol breach which resulted in bipolar iEGMs of one animal not being recorded correctly.

### Ex vivo tissue examination

Images of the gross pathological tissue sections were acquired with a camera set at a fixed distance (resolution: 200 pixels/in). Lesion depth and width measurements were made manually using Fiji^84^. The cardiac fixed tissue slab harboring the largest lesion area per ablation was forwarded for histological processing. After dehydration in a graded series of alcohol, specimens were embedded in paraffin, cut with a microtome set to 5 μm, sections were floated onto glass slides, and two serial sections were stained with Hematoxylin & Eosin (H&E) and with Masson’s trichrome. The glass slides were then digitally imaged with the TissueScope LE (Huron Digital Pathology, St. Jacobs, Canada) in brightfield mode at 20x magnification (0.4 μm/pixel resolution), exported as TIFF files and reviewed using Sedeen Viewer (Pathcore, Toronto, Canada).

### Statistical analysis/methods

Analysis of temporal trends and dose dependency across PFA lesions was performed using linear mixed effects (LME) models to account for differences between individual animals and other random effects (R version 4.2, R Foundation for Statistical Computing, Vienna, Austria)^85^. The R packages lme4 and emmeans were used for LME modelling and post-hoc tests respectively. Details of the models are described in the following.

Dose-dependence of lesion volumes from LGE cMR images acquired at different time points following ablation (Fig. 3a-c) were evaluated using a model of the form: *volume ~ voltage + (1|Animal ID*) where *Animal ID* represents the individual animal from which the data was collected.

The temporal trends in LGE-derived lesion volumes created using different dose schemes (Fig. 3d) were evaluated using the model: *Volume ~ Time + (1|Animal ID) + (1|Voltage) + (1|Ntrains).*

Trends in wall thickness measured over time after ablation with various dose schemes (Fig. 4b) were evaluated using a model of the form: *Wall Thickness ~ Time + (1|Dose).*

Several parameters related to the dose dependence of iEGMs were investigated. To evaluate the dose dependency of absolute peak-to-peak bipolar voltage, the model took the form: p-p *V*oltage ~ Time + Dose + (1|*Animal* ID/Lesion *ID*), where Dose was set to either PFA voltage or number of trains delivered (corresponding to the comparisons shown in the left and right panels in Fig. 5d). Similarly, the normalized peak-to-peak voltages (Fig. 5e) were evaluated using the model: *Relative p-p Voltage ~ Time + Dose + (1|Animal ID/Lesion ID)*. Dose-dependency of COI (Fig. 5f) was evaluated using the model: *COI ~ Time + Dose + (1|Animal ID/Lesion ID)*. The relative COI (Fig. 5g) was modelled as: *Relative COI ~ Dose + (1|Animal ID)*, with the simpler model chosen due to the reduced number of datapoints after normalization.

Temporal trends in COI were investigated, with absolute COI in different underlying tissue types where PFA dosing was consistent (Fig. 8d) modelled as: COI ~ Time*Tissue + (1|Animal ID/Lesion ID). The normalized COI in each tissue type (Fig. 8e) was modelled as: *Relative COI ~ Tissue + (1|Animal ID).* These temporal trends were compared in lesions created in healthy underlying myocardium, in both healthy and infarcted animals (Fig. 8f). Unipolar voltage was evaluated using the model: *p-p Voltage ~ Time + Animal Model + (1|Animal ID/Lesion ID*), where *Animal Model* indicates whether the individual animal was healthy or infarcted.

The correlation between the lesion dimensions from LGE cMRI and gross pathology (Fig. 3d) were assessed using linear regression (python 3.7, scipy package, stats module).

P-values less than 0.05 were considered statistically significant throughout this study.

## Electronic supplementary material

Below is the link to the electronic supplementary material.

Supplementary Material 1

## Data Availability

All data are available in the main text in the associated repository. . Raw data can be obtained from Medtronic pending a Material Transfer Agreement. Contact corresponding author DM.
