# Supplementary material for: Biophysics and electrophysiology of pulsed field ablation in normal and infarcted porcine cardiac ventricular tissue
Source: Sci Rep. 2024 Dec 30;14:32063. doi: 10.1038/s41598-024-83683-y (PMC11686391; doi:10.1038/s41598-024-83683-y)
Supplement: Supplementary file 1 — Supplementary Material 1 [file 41598_2024_83683_MOESM1_ESM.pdf]

Biophysics and Electrophysiology of Pulsed Field Ablation in Normal and Infarcted Porcine Cardiac Ventricular Tissue

Supplementary material

Damijan Miklavčič*[1], Atul Verma[2], Philippa R. P. Krahn[3,6], Jernej Štublar[1, 4], Bor Kos[1], Terenz Escartin[3,6], Peter Lombergar[1], Nicolas Coulombe[5], Maria Terricabras[3], Tomaž Jarm[1], Matej Kranjc[1], Jennifer Barry[3], Lars Mattison[5], Nicole Kirchhof[5], Daniel C. Sigg[5], Mark Stewart[5], Graham Wright[3,6]

Affiliations:
[1]University of Ljubljana, Faculty of Electrical Engineering, Trzaska 25, Ljubljana, Slovenia
[2]McGill University Health Centre, McGill University, Montreal, Canada
[3]Sunnybrook Research Institute, Toronto, Canada
[4]University Clinical Medical Centre, Department of Cardiology Cardiovascular Surgery, Ljubljana, Slovenia
[5]Medtronic, Minneapolis, MN, USA
[6]Department of Medical Biophysics, University of Toronto, Toronto, Canada

*Corresponding author: Damijan Miklavčič
damijan.miklavcic@fe.uni-lj.si

Acknowledgements: This study has been funded by Medtronic, and in part by Slovenian Research and Innovation Agency through P2-0249 grant, Canadian Institutes of Health Research grant 178299, and GE Healthcare.

The authors are grateful for histology work provided by the Biomarker Imaging Research Lab at Sunnybrook Research Institute, funded through the Ontario Institute for Cancer Research, Calder Sheagren from University of Toronto for MRI sequence support, and Tadej Kotnik from University of Ljubljana for a thorough reading of the manuscript.

Author contributions according to CRediT:

Conceptualization: DM, AV, BK, MS, GW
Data curation: PRPK, JŠ, TE, NC, GW
Formal analysis: PRPK, JŠ, BK, TE, PL, TJ, GW
Funding acquisition: DM, NC, DCS, GW
Investigation: DM, AV, PRPK, JŠ, BK, TE, PL, NC, MT, MK, JB, GW
Methodology: DM, AV, PRPK, JŠ, BK, TE, PL, MT, TJ, MK, LM, GW
Project administration: DM, NC, JB, GW
Resources: NC, JB, LM, DCS, MS, GW, CS
Software: TE, PL, CS
Validation: DM, TE
Visualization: DM, PRPK, JŠ, BK, TE, PL, TJ, MK
Writing—original draft: DM, AV, PRPK, BK, PL, TJ, MK
Writing—review & editing: DM, AV, JŠ, BK, TE, NC, MT, MK, JB, LM, NK, DCS, MS, GW

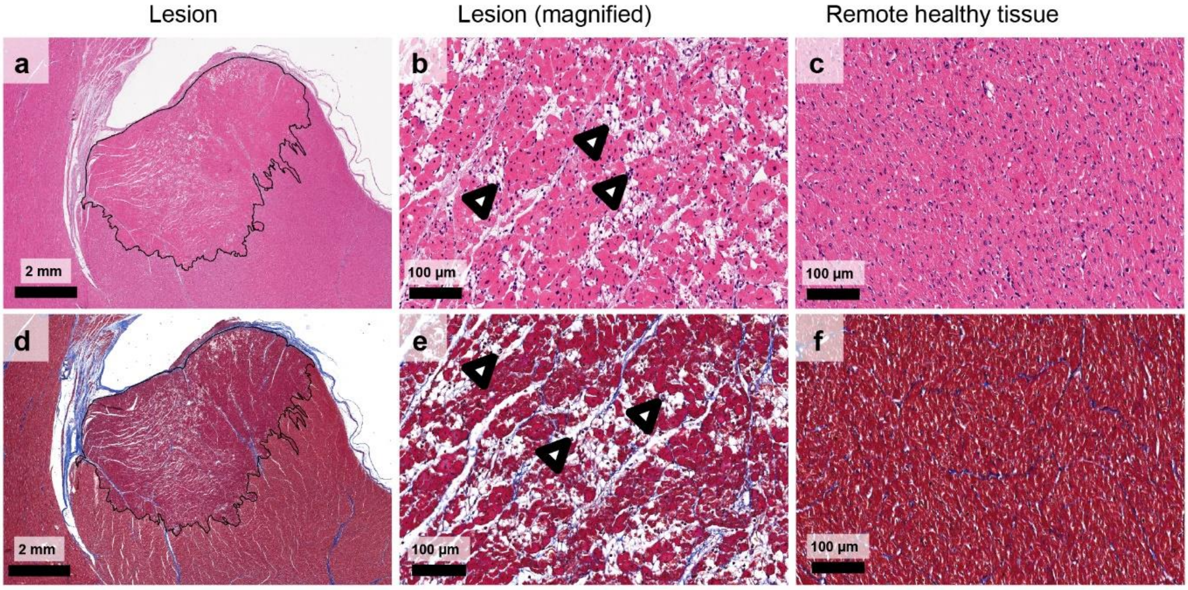

Supplementary Figure S1: Histological images of acute PFA lesion. Photomicrographs showing an acute, 7-hours old PFA lesion created using a dose of 1500V, 4 trains. Histology sections were stained with HE (**a-c**) or with Masson's trichrome (**d-f**). The lesion is outlined in black (**a,d**). Cardiomyocyte damage, interstitial edema, and scattered interstitial leukocytes (arrowheads) are shown in magnified sections from the center of the lesion (**b, e**) showcasing the acute tissue damage against examples from healthy remote regions (**c, f**).

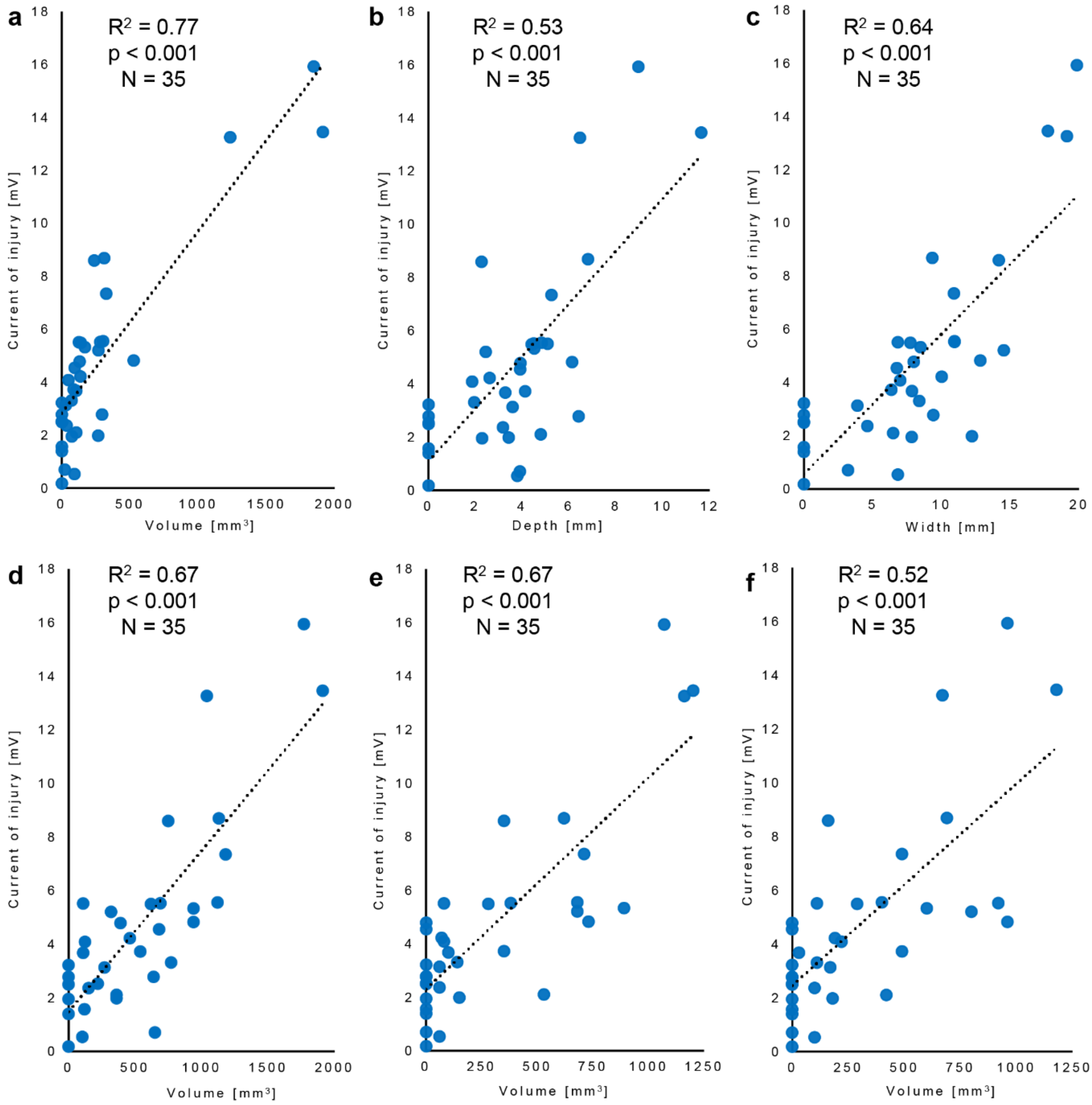

Supplementary Figure S2: Correlation between the current of injury (COI) parameter from unipolar iEGMs (bandwidth: 0.5 Hz – 500 Hz) and various measures of lesion size. Correlation coefficient (unadjusted) and the statistical significance of the linear fit are reported). (**a**-**c**) correlation to gross pathology data. (**a**) estimated volume (from depth and width of lesion, using the half-ellipsoid formula); (**b**) depth of lesion; (**c**) width of lesion. (**d**-**f**) correlation to LGE cMRI-measured volume at 3 different post-ablation time points. (**d**) 24 hours; (**e**) 7 days; (**f**) 6 weeks.

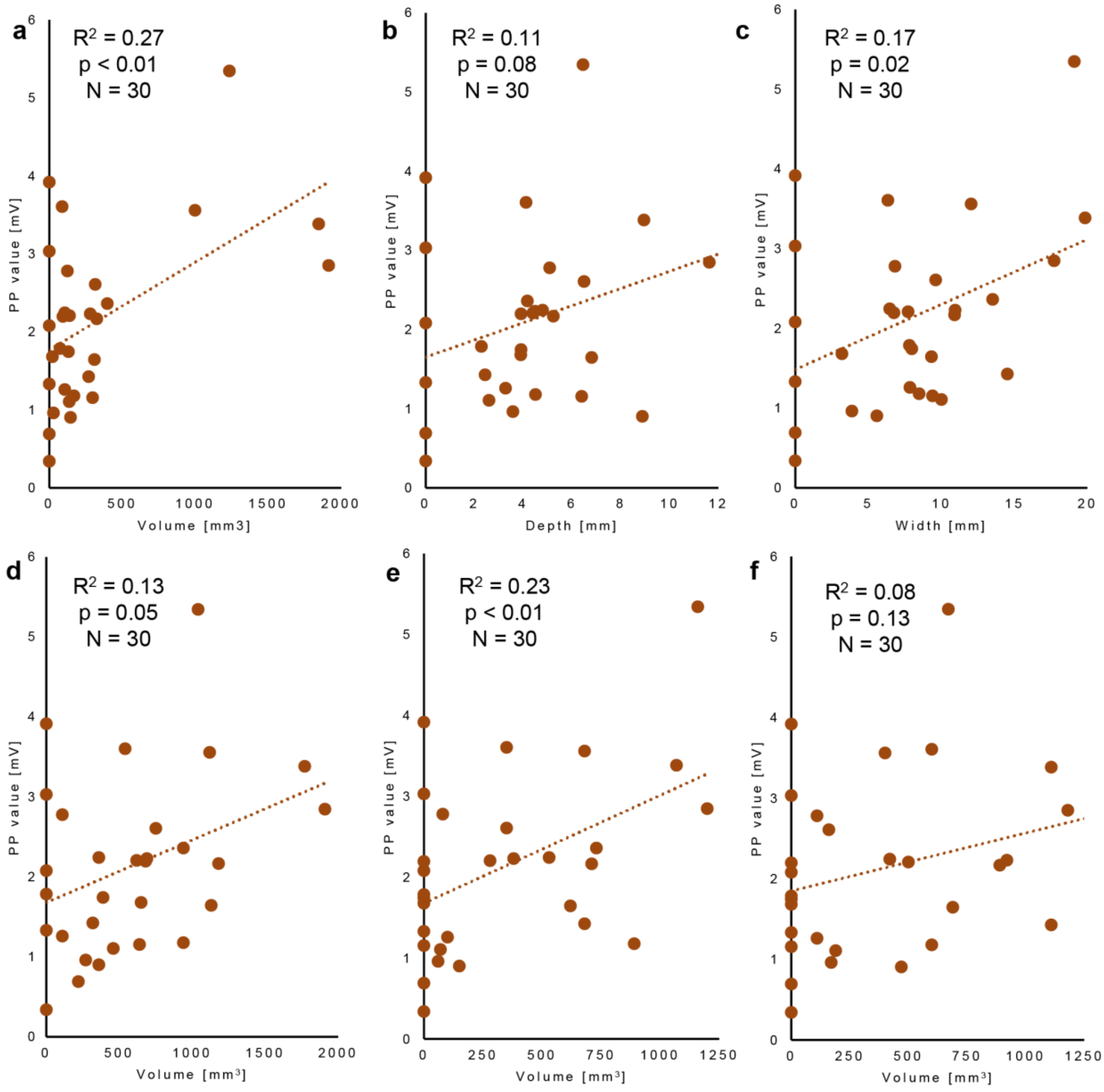

Supplementary Figure S3: Correlation between the peak-to-peak amplitude of bipolar iEGMs (bandwidth: 30 Hz – 500 Hz) and various measures of lesion size. The correlation coefficient (unadjusted) and the statistical significance of the linear fit are reported). (**a-c**) correlation to gross pathology data. (**a**) estimated volume (from depth and width of lesion, using the half-ellipsoid formula); (**b**) depth of lesion; (**c**) width of lesion. (**d-f**) correlation to LGE cMRI-measured volume at 3 different post-ablation time points. (**d**) 24 hours; (**e**) 7 days; (**f**) 6 weeks).

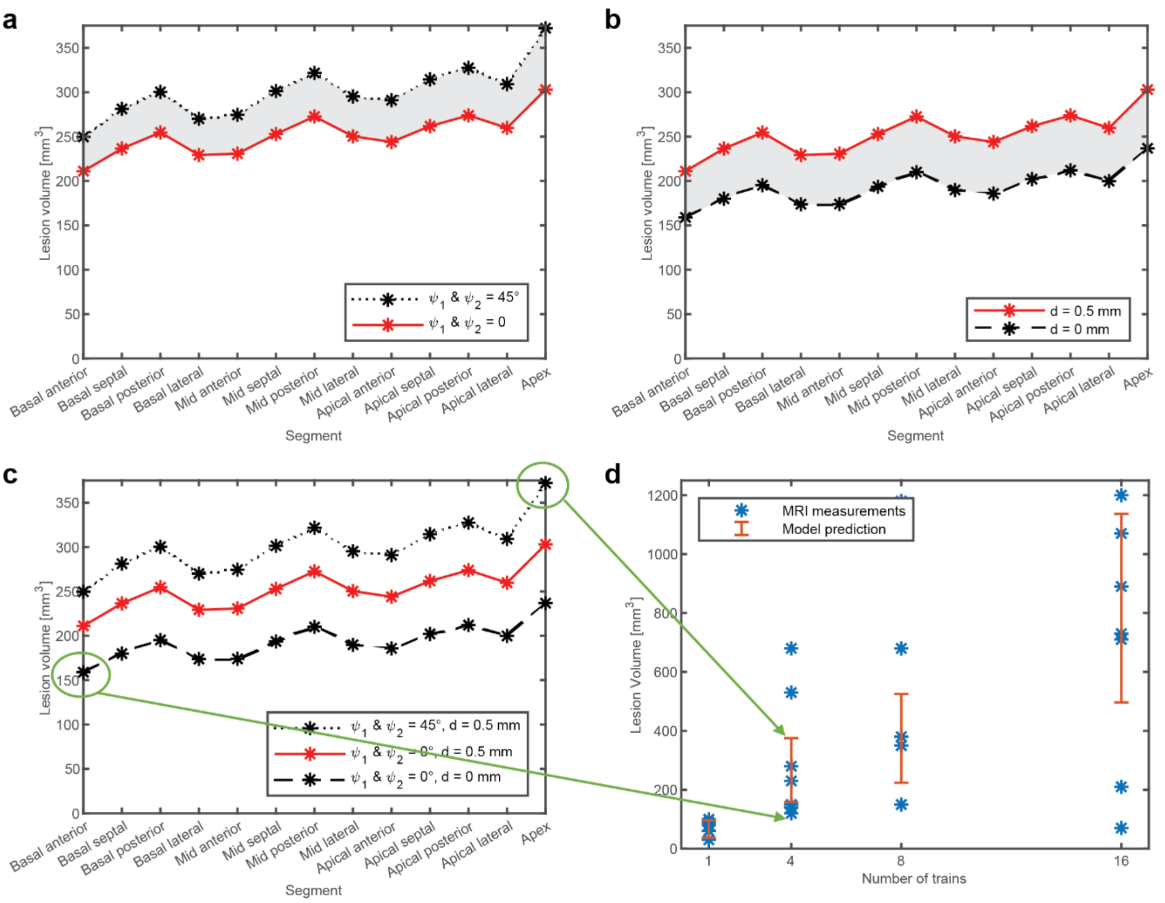

Supplementary Figure S4: Effect of catheter insertion depth, angle, and lesion location on computed lesion volume. (**a**) Effect of catheter angle on the calculated lesion volume in different LV segments for the calculated 4 train 7D LGE cMRI LET (520 V/cm). Catheter insertion depth was fixed at 0.5 mm. ψ_1 represents the inclination of the catheter shaft in the up-down direction (base-apex) and ψ_2 represents the rotation of the catheter around the z axis of the model, where $\psi_1 \,\&\, \psi_2 = 0°$ means the catheter is positioned perpendicular to the myocardial wall. (**b**) Effect of catheter insertion depth on the calculated lesion volume in different LV segments for the calculated 4 train 7D LGE cMRI LET (520 V/cm). The catheter was perpendicular to the myocardial wall. (**c**) Combination of catheter angle and insertion depth which results in minimum and maximum calculated lesion volume (black) for the calculated 4 train 7D LGE cMRI LET (520 V/cm), and also the combination we used in the model to find the LET (red). (**d**) Comparison of measured lesion volumes (7D LGE cMRI LET) for 1, 4, 8 and 16 trains (indicated by *) with the uncertainty of lesion volumes when accounting for the variability in catheter angles, insertion depth and location. Error bars represent the minimum and maximum calculated lesion volume for each 7D LGE cMRI LET (1, 4, 8, 16 trains), that we obtain by changing lesion location (1-13), catheter insertion depth (0-0.5mm) and angle (0- 45°).

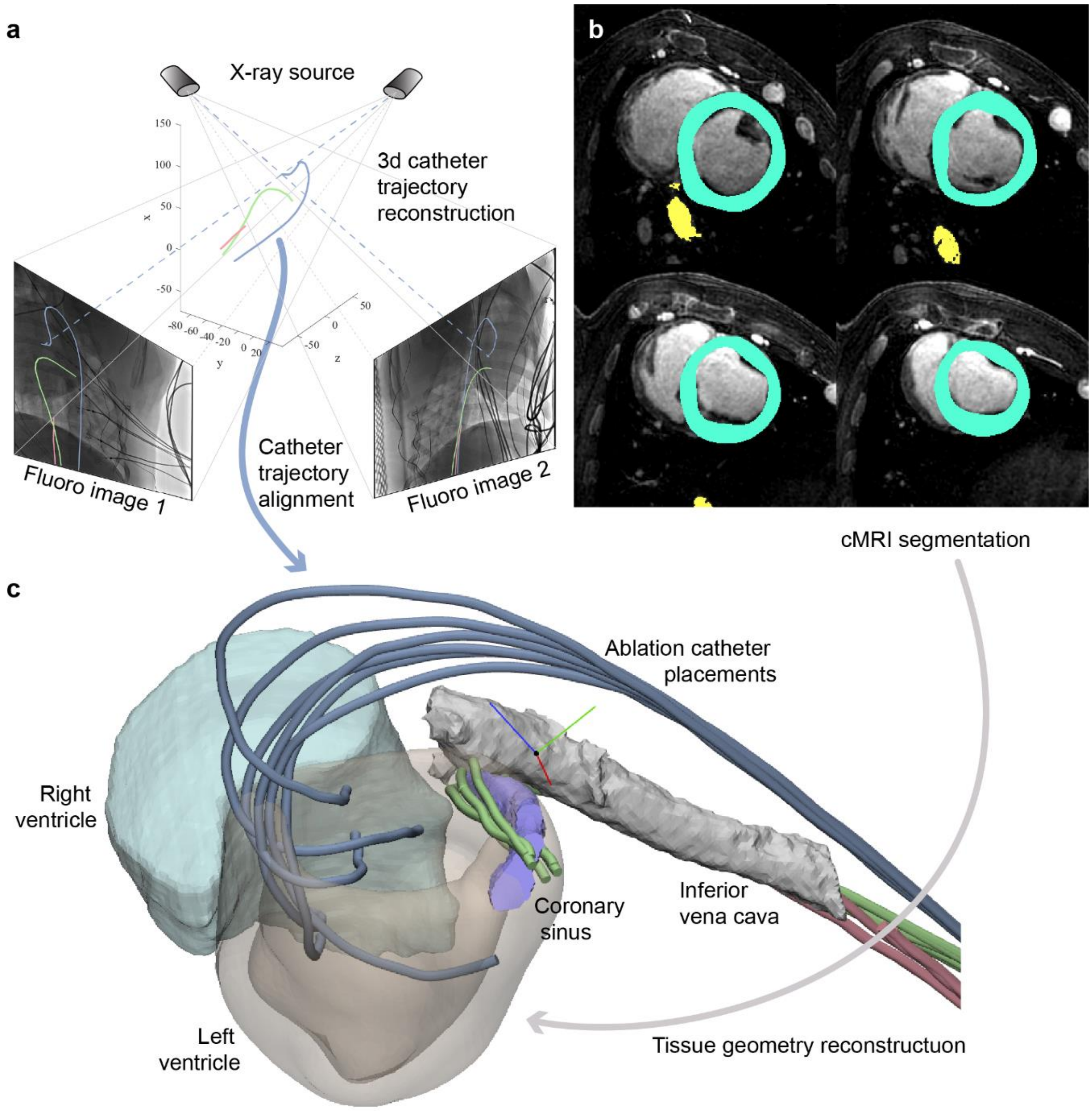

Supplementary Figure S5: Catheter location reconstruction process. (**a**) Trajectories of all catheters are marked from fluoroscopy images in diastolic phase. Based on known imager rotations, the intersection between rays extending from source to catheter image on detector is found. This forms trajectories in 3D space. Image generated using Matlab (Mathworks, Natick, MA) and workflow illustrated using Adobe Illustrator (Adobe, San Jose, CA) (**b**) cMRI images were segmented usimg Mimics (Materialise, Leuven, Belgium) and a 3D reconstruction of the ventricles, the inferior vena cava and the coronary sinus were created. (**c**) The ensemble of the catheter trajectories was manually aligned with the landmarks identified in imaging to find locations of the ablation catheter placements using 3-Matic (Materialise, Leuven, Belgium).

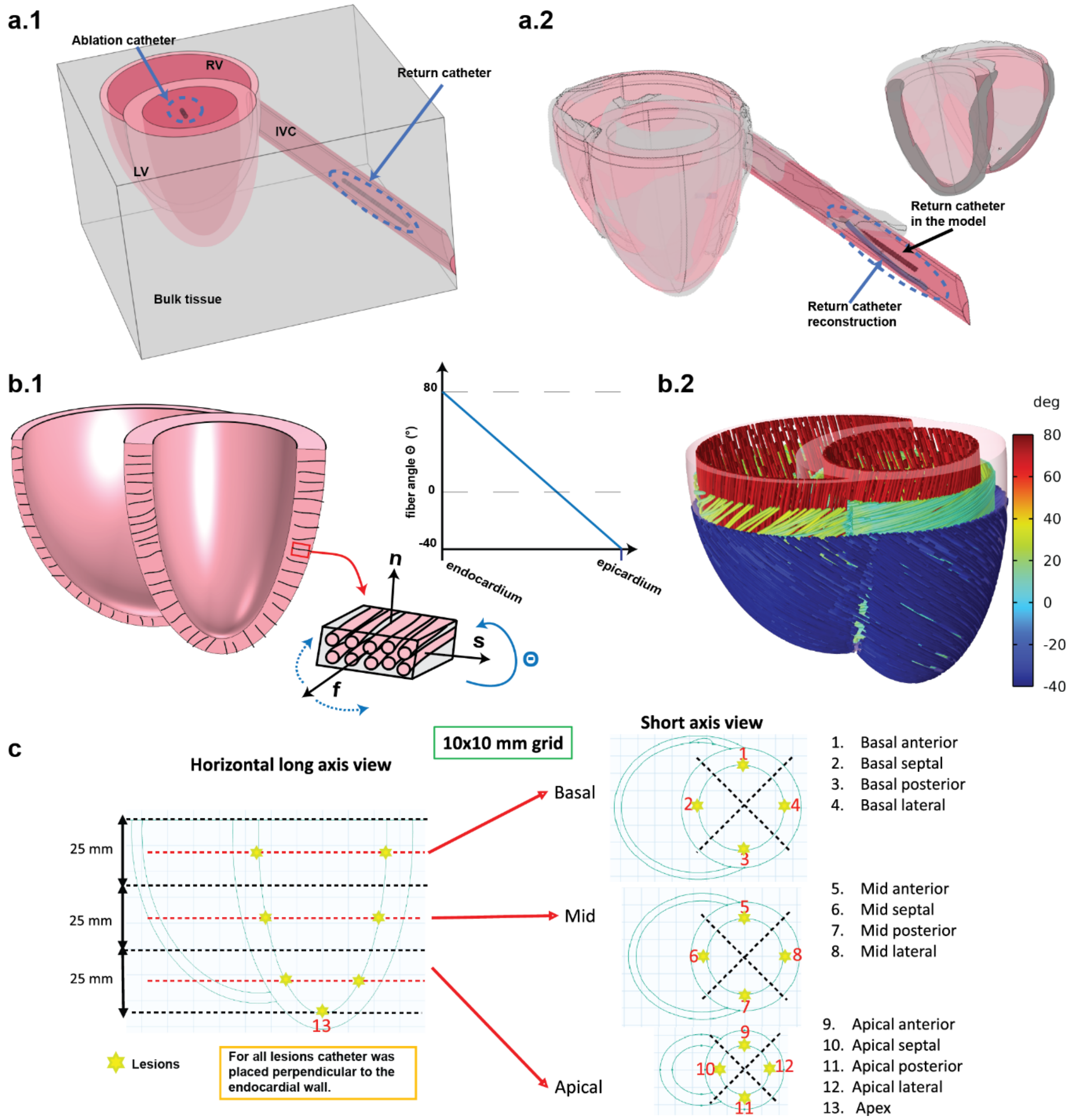

Supplementary Figure S6: Construction of the schematic numerical model. (**a1**) 3D-geometry of the numerical model built and imaged using COMSOL Multiphyisics. The model includes: left ventricle (LV), right ventricle (RV), inferior vena cava (IVC), ablation catheter, return catheter and bulk tissue. (**a2**) Example of alignment between the schematic biventricular model built in COMSOL with the imported cMRI-based model of the ventricles and IVC complex (colored gray) and alignment of the return catheter in the model (colored black) with the fluoroscopy-based catheter reconstruction (colored blue). (**b1**) Schematic representation of myocardial structure, which was modeled with three orthogonal directions: fiber direction (**f**), sheet direction (**s**), and normal to sheet direction (**n**). To account for change in fiber direction from endocardium to epicardium in the model, we rotated the fiber direction (f) by and angle Θ around the sheet direction (**s**), which was approximated with the transmural direction. A linear variation of the fiber angle across the wall was assumed with a fiber inclination of 80° at the endocardium and -40° at the epicardium. (**b2**) Streamline plot of the myocardial fiber direction (**f**), with different colors depicting different inclination of the fiber across the myocardial wall. (**c**) Segmentation of the left ventricle (13 segments) and position of the ablation catheter.

Supplementary Table S1: Material properties used in the numerical model

Material	Physical property	Value	Reference
Myocardium	$\sigma_{\parallel}$ - electrical conductivity parallel to the fibers	$0.5\,\frac{S}{m}$	(Kos et al. 2023)
	$\sigma_{\perp}$ - electrical conductivity perpendicular to the fibers	$0.375\,\frac{S}{m}$	(Kos et al. 2023)
	ρ_{myo} - density	$1081\,\frac{kg}{m^3}$	(Hasgall et al. 2022), heart muscle
	C_{myo} - thermal capacity	$3686\,\frac{J}{kgK}$	(Hasgall et al. 2022), heart muscle
	k_0 - thermal conductivity at 37°C	$0.56\,\frac{W}{mK}$	(Hasgall et al. 2022), heart muscle
	A - frequency factor (Arrhenius)	$2.94 \cdot 10^{39}\,s^{-1}$	(Pearce 2011)
	ΔE - activation energy	$2.596 \cdot 10^{5}\,\frac{J}{mol}$	(Pearce 2011)
Scar	σ_{scar} - electrical conductivity	$1\,\frac{S}{m}$	(Cinca et al. 1998; Salazar et al. 2004)
Blood	σ_{blood} - electrical conductivity	$0.7\,\frac{S}{m}$	(Hasgall et al. 2022), blood 10 kHZ
	ρ_{blood} - density	$1050\,\frac{kg}{m^3}$	(Hasgall et al. 2022), blood
	C_{blood} - thermal capacity	$3617\,\frac{J}{kgK}$	(Hasgall et al. 2022), blood
	k_{blood} - thermal conductivity	$0.52\,\frac{W}{mK}$	(Hasgall et al. 2022), blood
	μ_{blood} - dynamic viscosity	$0.0035\,\frac{kg}{ms}$	(Doost et al. 2016)
Bulk tissue	σ_{bulk} - electrical conductivity	$0.38\,\frac{S}{m}$	Original value determined from current measurements
	ρ_{bulk} - density	$1027\,\frac{kg}{m^3}$	(Hasgall et al. 2022), connective tissue
	C_{bulk} – thermal capacity	$2372\,\frac{J}{kgK}$	(Hasgall et al. 2022), connective tissue
Pt/Ir electrodes	$\rho_{Pt/Ir}$ - density	$21400\,\frac{kg}{m^3}$	COMSOL Material Library, Pt (solid)
	$C_{Pt/Ir}$ – thermal capacity	$133\,\frac{J}{kgK}$	COMSOL material library, Pt (solid)
	$k_{Pt/Ir}$ – thermal conductivity	$31\,\frac{W}{mK}$	(Sun et al. 2023)

Additional data is available in a public repository: 10.6084/m9.figshare.25894936

The repository contains two files. Please find below the description of the data.

Excel spreadsheet DataS1.xlsx This is the data used to for all statistics and for the generation of figures in the main manuscript and in of the supplementary figures.

Supplementary Table S2:
Description of the data table **DataS1.xlsx** available in the Figshare repository. For reference, the data legends is included here.

Data category	Header	Description
**Data point**	**Pig**	Animal ID
	**Lesion**	Lesion ID
**Protocol information**	**Voltage**	Applied PFA Voltage (V)
	**Trains**	# Pulse Trains Delivered
	**Targeted_Location**	Targeted Location during pretreatment plan
	**Rec_length**	Duration of iEGM Recording Duration Post-Ablation period
	**MR_13_Segment_Location**	Segment # Derived From MRI (13-Segment Model of the LV)
**Lesion Dimensions, Gross Pathology**	**GP_depth**	Depth (mm)
	**GP_width**	Width (mm)
	**GP_vol_formula**	Estimated Volume (mm^3) based on formula d*w^2*PI/6
**Lesion Dimensions, 24h MRI**	**MR24h_depth**	Depth (mm)
	**MR24h_width**	Width (mm)
	**MR24h_vol_formula**	Estimated Volume (mm^3) based on formula d*w^2*PI/6
	**MR24h_vol**	Measured Volume (cm^3) from segmented MRI images
**Lesion Dimensions, 7d MRI**	**MR7d_vol**	Measured Volume (cm^3) from segmented MRI images
	**MR7d_depth**	Depth (mm)
	**MR7d_width**	Width (mm)
**Lesion Dimensions, 24d MRI**	**MR24d_vol**	Measured Volume (cm^3) from segmented MRI images
	**MR24d_depth**	Depth (mm)
	**MR24d_width**	Width (mm)
**Lesion Dimensions, 6w MRI**	**MR6w_vol**	Measured Volume (cm^3) from segmented MRI images
	**MR6w_depth**	Depth (mm)
	**MR6w_width**	Width (mm)
**Infarct properties**	**InfarctedAnimal**	Boolean value if animal was infarcted or not

	**Targeted_tissue**	What tissue type was targeted (Healthy, infarcted, border)
	**MR_Center_of_Lesion**	Actual Underlying Tissue (at Lesion Center in MRI)
	**GP_whole_lesion**	Actual Underlying Tissue (defined using lesion edges in gross pathology)
	**GP_approx_scar_dist**	Distance From Scar (mm; estimated using gross pathology)
**Lethal Electric Field Threshold (V/cm)**	**LET_24H**<br>**LET_7D**<br>**LET_6W**	Calculated using MRI at 24h<br>Calculated using MRI at 7d<br>Calculated using MRI at 6w
**iEGM Peak-to-Peak Amplitude (mV)**	**iEGM_PP_pre**<br>**iEGM_PP_30s**<br>**iEGM_PP_3.5m**	Pre-Ablation<br>30s Post-Ablation<br>3.5min Post-Ablation
**Relative iEGM P-P Amplitude**	**REL_iEGM_PP_30s**<br>**REL_iEGM_PP_3.5m**	30s Post-Ablation Vs Pre-Ablation<br>3.5min Post-Ablation Vs Pre-Ablation
**Current of Injury (mV)**	**CoI_pre**<br>**CoI_30s**<br>**CoI_3.5m**	Pre-Ablation<br>30s Post-Ablation<br>3.5min Post-Ablation (Healthy animals) and 5 min Post-Ablation (Infarcted animals)
**Relative Current of Injury**	**REL_CoI**	Relative value of Current of Injury parameter between post and pre-ablation

`Miklavcic_et_al_Schematic_PFA_ventricle_model.mph`

Numerical model file from COMSOL Multiphysics. Created in version 6.2. This numerical model contains all the simulation settings used for determining the lethal electric field.

References supplementary material

Cinca, J., M. Warren, A. Rodríguez-Sinovas, M. Tresànchez, A. Carreño, R. Bragós, O. Casas, A. Domingo, and J. Soler-Soler. 1998. “Passive Transmission of Ischemic ST Segment Changes in Low Electrical Resistance Myocardial Infarct Scar in the Pig.” *Cardiovascular Research* 40 (1): 103–12. https://doi.org/10.1016/s0008-6363(98)00145-x.

Doost, Siamak N., Dhanjoo Ghista, Boyang Su, Liang Zhong, and Yosry S. Morsi. 2016. “Heart Blood Flow Simulation: A Perspective Review.” *BioMedical Engineering OnLine* 15 (1): 101. https://doi.org/10.1186/s12938-016-0224-8.

Hasgall, P A, F Di Gennaro, C Baumgartner, E Neufeld, B Lloyd, M C Gosselin, D Payne, A Klingenböck, and N Kuster. 2022. “IT’IS Database for Thermal and Electromagnetic Parameters of Biological Tissues.,” no. Version 4.1 (February). https://doi.org/10.13099/VIP21000-04-1.

Kos, Bor, Lars Mattison, David Ramirez, Helena Cindrič, Daniel C. Sigg, Paul A. Iaizzo, Mark T. Stewart, and Damijan Miklavčič. 2023. “Determination of Lethal Electric

Field Threshold for Pulsed Field Ablation in Ex Vivo Perfused Porcine and Human Hearts." *Frontiers in Cardiovascular Medicine* 10:1160231 (June). https://doi.org/10.3389/fcvm.2023.1160231.

Pearce, John. 2011. "Mathematical Models of Laser-Induced Tissue Thermal Damage." *International Journal of Hyperthermia* 27 (8): 741–50. https://doi.org/10.3109/02656736.2011.580822.

Salazar, Y., R. Bragos, O. Casas, J. Cinca, and J. Rosell. 2004. "Transmural versus Nontransmural in Situ Electrical Impedance Spectrum for Healthy, Ischemic, and Healed Myocardium." *IEEE Transactions on Biomedical Engineering* 51 (8): 1421–27. https://doi.org/10.1109/TBME.2004.828030.

Sun, Hongsheng, Haoran Chen, Yu Chen, Chaoqun Wei, Qing Wang, Youyu Gan, Yanwu Qi, and Jianli Wang. 2023. "Thermal and Electrical Conductivities of Platinum/Iridium Microwires." *Journal of Electronic Materials* 52 (3): 2185–92. https://doi.org/10.1007/s11664-022-10190-z.
